# Under phosphate starvation conditions, Fe and Al trigger accumulation of the transcription factor STOP1 in the nucleus of Arabidopsis root cells

**DOI:** 10.1111/tpj.14374

**Published:** 2019-06-04

**Authors:** Christian Godon, Caroline Mercier, Xiaoyue Wang, Pascale David, Pierre Richaud, Laurent Nussaume, Dong Liu, Thierry Desnos

**Affiliations:** ^1^ Laboratoire de Biologie du Développement des Plantes, Commissariat à l'Énergie Atomique et aux Énergies Alternatives UMR7265 (CEA, Aix‐Marseille Université, CNRS) Saint Paul‐Lez‐Durance F‐13108 France; ^2^ Ministry of Education Key Laboratory of Bioinformatics Center for Plant Biology School of Life Sciences Tsinghua University Beijing 100084 China; ^3^ Laboratoire de Bioénergétique et Biotechnologie des Bactéries et Microalgues Commissariat à l'Énergie Atomique et aux Énergies Alternatives UMR7265 (CEA, Aix‐Marseille Université, CNRS) Saint Paul‐Lez‐Durance F‐13108 France

**Keywords:** STOP1, ALMT1, ALS3, phosphate, iron, aluminum

## Abstract

Low‐phosphate (Pi) conditions are known to repress primary root growth of Arabidopsis at low pH and in an Fe‐dependent manner. This growth arrest requires accumulation of the transcription factor STOP1 in the nucleus, where it activates the transcription of the malate transporter gene *ALMT1*; exuded malate is suspected to interact with extracellular Fe to inhibit root growth. In addition, ALS3 – an ABC‐like transporter identified for its role in tolerance to toxic Al – represses nuclear accumulation of STOP1 and the expression of *ALMT1*. Until now it was unclear whether Pi deficiency itself or Fe activates the accumulation of STOP1 in the nucleus. Here, by using different growth media to dissociate the effects of Fe from Pi deficiency itself, we demonstrate that Fe is sufficient to trigger the accumulation of STOP1 in the nucleus, which, in turn, activates the expression of *ALMT1*. We also show that a low pH is necessary to stimulate the Fe‐dependent accumulation of nuclear STOP1. Furthermore, pharmacological experiments indicate that Fe inhibits proteasomal degradation of STOP1. We also show that Al acts like Fe for nuclear accumulation of STOP1 and *ALMT1* expression, and that the overaccumulation of STOP1 in the nucleus of the *als3* mutant grown in low‐Pi conditions could be abolished by Fe deficiency. Altogether, our results indicate that, under low‐Pi conditions, Fe^2/3+^ and Al^3+^ act similarly to increase the stability of STOP1 and its accumulation in the nucleus where it activates the expression of *ALMT1*.

## Significance Statement

Low‐phosphate, low‐pH and excess aluminum are three major stresses inhibiting root growth. The Arabidopsis transcription factor STOP1 has a major role in root growth under these different stresses, but how STOP1 is regulated is not known. Here we show that Fe –instead of low‐phosphate– and Al promote the accumulation of STOP1 in root nuclei, in a low‐pH‐dependent manner. This work unveils an important regulatory step in the response to Al and Fe stresses.

## Introduction

In many plant species, phosphate (Pi) deficiency (–Pi) alters root growth and architecture, promoting top‐soil foraging: the growth of the primary root is reduced whereas lateral root emergence and growth is stimulated toward a more horizontal direction. Combined, these responses result in root systems that explore relatively better the upper soil horizons where Pi is more concentrated (Lynch and Brown, 2001).

The Arabidopsis primary root stops growing when its apex encounters the –Pi substratum (Svistoonoff *et al*., 2007). Split‐root and feeding experiments have shown that this inhibition of root growth does not correlate with Pi accumulated inside the root (Thibaud *et al*., 2010). Instead, it depends on the concentration of Pi in the growth medium. This phenomenon was described as the so‐called –Pi local response, in opposition to the –Pi systemic responses that are governed by the Pi concentration inside the plant tissues (Puga *et al*., 2017). While the –Pi systemic response is mainly controlled by the two master regulatory genes *PHOSPHATE STARVATION RESPONSE 1* (*PHR1*) and *PHR1‐like 1* (*PHL1*), the –Pi local response is not (Balzergue *et al*., 2017). This local growth inhibition response critically depends on the Fe content of the growth medium (Svistoonoff *et al*., 2007; Ward *et al*., 2008; Muller *et al*., 2015), and Fe accumulates in the root tip (Muller *et al*., 2015; Balzergue *et al*., 2017; Mora‐Macias *et al*., 2017).

Due to its chemical nature Pi can form complexes with metallic cations such as Fe^2/3+^ and Al^3+^ (Hinsinger, 2001). This has important consequences for the mobility and bioavailability in soil of Pi, Fe and Al as well as for their homeostasis in plants (Misson *et al*., 2005; Hirsch *et al*., 2006; Bouain *et al*., 2014; Briat *et al*., 2015). In the medium, reflecting the antagonistic Fe–Pi interactions, the Pi/Fe ratio is important: the lower the ratio the higher the inhibition of root growth, and when the nutrient solution lacks Fe root growth is no longer inhibited (Svistoonoff *et al*., 2007; Ward *et al*., 2008; Muller *et al*., 2015).

During the last decade, major advances have contributed to a better understanding of the cellular events underlying the ‘local' response in Arabidopsis. When exposed to low external Pi (i.e. a low Pi/Fe ratio), a short primary root results from a combination of rapid as well as long‐term cellular responses at the root tip. A few hours, if not minutes, after the root tip encounters a Pi‐depleted zone, cell elongation decreases. This is correlated with the deposition of Fe and the accumulation of reactive oxygen species (ROS) in the cell wall of the stem cell niche (SCN) and the elongation zone (EZ), and with a peroxidase‐dependent stiffening of the cell wall of the EZ.

In the long term (hours to days), callose accumulates in the cell wall of the root apical meristem (RAM) and EZ, this occludes plasmodesmata and restricts cell‐to‐cell trafficking. Progressively, root cell proliferation ceases until exhaustion of the RAM by the on‐going cell differentiation (Muller *et al*., 2015; Balzergue *et al*., 2017; Mora‐Macias *et al*., 2017).

Genetic and molecular analysis has identified several proteins governing root growth inhibition under –Pi. Identified as a major quantitative trait locus, *LOW PHOSPHATE ROOT 1* (*LPR1*) encodes a cell wall multi‐copper oxidase with ferroxidase activity (Reymond *et al*., 2006; Svistoonoff *et al*., 2007; Ticconi *et al*., 2009; Muller *et al*., 2015). LPR1 has a critical role in the growth arrest because loss‐of‐function mutations in *lpr1* desensitize roots to –Pi. In particular, they accumulate a reduced amount of Fe in their tips. Upon extended Pi deprivation, the LPR1‐dependent accumulation of Fe promotes exhaustion of the RAM and differentiation by downregulation of the root patterning transcription factors SHORT ROOT (SHR) and SCARECROW (SCR). This regulation operates via increased expression of CLAVATA 4 (CLV4), a secreted peptide detected by the plasma membrane receptors CLV2 and CLV2/PEP1 RECEPTOR 2 (PEPR2) (Gutiérrez‐Alanís *et al*., 2017). A higher level of regulation coupling LPR1 with brassinosteroid (BR) signaling pathways has recently been unveiled. In particular, the root growth of seedlings with the constitutively active *bzr1‐D* mutation (*BZR1* for *BRASSINAZOLE RESISTANT1*) is unrepressed in –Pi and expression of *LPR1* is reduced (Singh *et al*., 2014, 2018). In addition, in the wild type (WT), Fe enhances the accumulation of the BR‐signaling inhibitor BKI1 (BRASSINSOSTEROID KINASE INHIBITOR1), thereby closing a feedback regulatory loop between LPR1 activity and BR signaling.

The *PHOSPHATE DEFICIENCY RESPONSE 2* (*PDR2*) gene was identified in the local response (Ticconi *et al*., 2004). PDR2 is the only Arabidopsis P5‐type ATPase; it is located in the endoplasmic reticulum and its substrate is not yet known (Ticconi *et al*., 2009). Compared with *lpr1* mutations, a mutation inactivating *PDR2* confers an opposite effect (i.e. overaccumulation of Fe; Muller *et al*., 2015). The *lpr1* mutations are epistatic over *pdr2*, indicating a functional interaction between LPR1 and PDR2.

A large‐scale forward genetics screen for seedlings with a primary root less sensitive to –Pi inhibition identified several *stop1* and *almt1* mutants (in addition to *lpr1*) (Balzergue *et al*., 2017). SENSITIVE TO PROTON RHIZOTOXICITY1 (STOP1) is a C_2_H_2_ zinc finger transcription factor necessary for the expression of *ALUMINUM ACTIVATED MALATE TRANSPORTER 1* (*ALMT1*), encoding a plasma membrane transporter exuding malate (Hoekenga *et al*., 2006; Iuchi *et al*., 2007). While the *stop1* and *almt1* mutants have reduced accumulation of Fe in the EZ, they still accumulate large amounts of Fe in the SCN (Balzergue *et al*., 2017; Wang *et al*., 2019), although others observed reduced accumulation of Fe in the SCN (Mora‐Macias *et al*., 2017). This is correlated with some remaining root inhibition upon long‐term Pi deprivation, presumably mediated by Fe‐dependent RAM differentiation and exhaustion. These observations show that the STOP1–ALMT1 module is mainly involved in the inhibition of cell elongation whereas the LPR1–PDR2 module influences all aspects of the local response. Supporting independent genetic regulation of these two modules, the *lpr1* mutation does not alter the expression of *ALMT1* and, reciprocally, *stop1* mutants display WT expression of *LPR1*. It seems that, at least in the EZ, the convergence point of these two modules is in the apoplast compartment where the ALMT1‐exuded malate somehow helps Fe to participate in the generation of ROS.

In the root tip, Pi deprivation enhances the transcript level of *ALMT1* but not of *STOP1*, suggesting post‐translational regulation of STOP1 protein. Indeed, the –Pi condition stimulates the accumulation of STOP1 in the nucleus (Balzergue *et al*., 2017). This nuclear accumulation of STOP1 therefore represents an important control step in this pathway for which a first, and unexpected, regulatory component has been identified recently. A genetics screen for mutants hypersensitive to –Pi‐induced inhibition of root growth retrieved an *als3* mutant. The ALS3 protein (ALUMINUM SENSITIVE 3) interacts with STAR1 (SENSITIVE TO ALUMINUM RHIZOTOXICITY 1) to form a putative ATP‐binding cassette (ABC) transporter complex located in the tonoplast (Dong *et al*., 2017). Consistently, in –Pi, the *star1* mutant behaves like *als3*. Interestingly, the root hypersensitivity of *als3* and *star1* mutants correlates with higher accumulation of STOP1 in root nuclei and overexpression of *ALMT1* in the root tip. By contrast, in –Pi, overexpression of the ALS3–STAR1 fusion protein represses the accumulation of STOP1 in the nucleus and improves root growth of the WT. Moreover, a suppressor screen of *als3* identified *stop1* and *almt1* mutants (as well as *lpr1*) (Wang *et al*., 2019). Taken together, these results show that, in combination, ALS3 and STAR1 attenuate root growth inhibition in –Pi by repressing the accumulation of STOP1 in the nucleus. This led us to hypothesize that ALS3–STAR1 depletes an unknown cytosolic compound (toward the vacuole) that enhances the accumulation of STOP1 in the nucleus (Wang *et al*., 2019).

Before the discovery of their implication in the response to –Pi, STOP1, ALMT1, ALS3 and STAR1 were all known for their major role in the resistance of Arabidopsis to toxic Al. Loss‐of‐function mutations in any of these genes severely impair root growth in the presence of Al^3+^ (Larsen *et al*., 2005; Hoekenga *et al*., 2006; Iuchi *et al*., 2007; Huang *et al*., 2010). It is therefore tempting to deduce that in –Pi conditions, Fe^2/3+^ is the metallic ion responsible for triggering nuclear accumulation of STOP1, thereby participating in repression of root growth in –Pi.

In this work, we found that growth conditions with limited Pi allowed us to distinguish the effect of Fe from –Pi *per se*. This enabled us to compare the effect of Fe with Al on STOP1 and ALMT1. Our results demonstrate that Fe, as well as Al, triggers the accumulation of STOP1 in the nucleus and the expression of *ALMT1*.

## Results

We previously showed that low‐Pi conditions stimulate the expression of the *ALMT1* gene, and this stimulation relies of the transcription factor STOP1 (Balzergue *et al*., 2017). In addition, under low Pi, omission of Fe from the nutrient solution prevents the arrest of root growth, showing that Fe is essential for this growth response (Svistoonoff *et al*., 2007; Ward *et al*., 2008; Muller *et al*., 2015; Dong *et al*., 2017; Mora‐Macias *et al*., 2017). However, the –Fe conditions previously tested did not completely suppress the expression of *ALMT1*.

### Iron, but not Pi deficiency *per se*, induces expression of *ALMT1*

**Figure 1:**
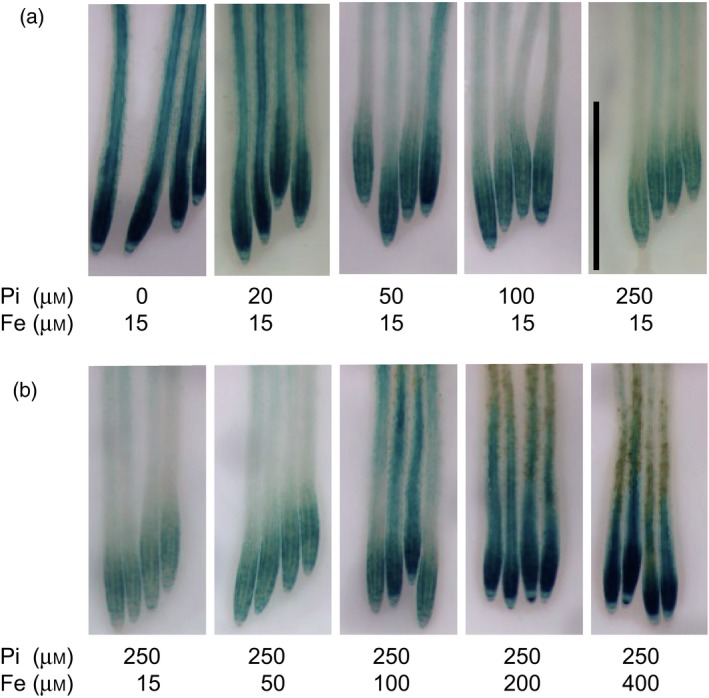
Antagonistic interactions of phosphate (Pi) and Fe on the expression of *pALMT1::GUS*. (a) Effect of the Pi concentration on GUS staining when Fe is 15 μm. (b) Effect of the Fe concentration on GUS staining when Pi is 250 μm. Seedlings were grown for 3 days on a pH 6.7 medium not supplemented with Pi and Fe, and transferred for 24 h to a pH 5.5 medium supplemented with the indicated concentrations of Pi and Fe, before GUS staining. Bar = 1 mm.

We used the *pALMT1::GUS* (*GUS*,* uidA* gene encoding β‐glucuronidase) construct to investigate the role of Fe because this visual reporter sensitively allows to test the activity of STOP1 signaling in the root tip (Balzergue *et al*., 2017). We first tested the influence of the Pi/Fe ratio on the expression of *ALMT1*. Seedlings were first grown for 3 days on a –Pi medium without added Fe. To prevent the expression of *ALMT1* (and thus the accumulation of the GUS protein) during this pre‐culture, the growth medium was buffered at pH 6.7. Indeed, at a pH around neutrality we observed no or very little GUS staining in the root tip of *pALMT1::GUS* seedlings (Balzergue *et al*., 2017; Figure S1 in the online Supporting Information). Seedlings were then transferred from this pre‐culture condition to media differing in their Fe:Pi ratio, at pH 5.5. Twenty‐four hours after transfer, the seedlings were stained for GUS activity. In a medium supplemented with 15 μm of Fe, and without Pi added, there is a strong expression of *ALMT1* (Figure 1a). Increasing the Pi content reduces the expression of *ALMT1*. However, at the highest Pi concentration tested (250 μm), the expression of *ALMT1* was still induced. This result confirms that *ALMT1* expression is higher in –Pi than in +Pi conditions. However, increasing the Fe content in a medium containing 250 μm Pi increases the expression of *ALMT1* (Figure 1b). This confirms the result of Muller *et al*. (2015) and suggests that Fe, instead of Pi deficiency *per se*, stimulates the expression of *ALMT1*.

**Figure 2:**
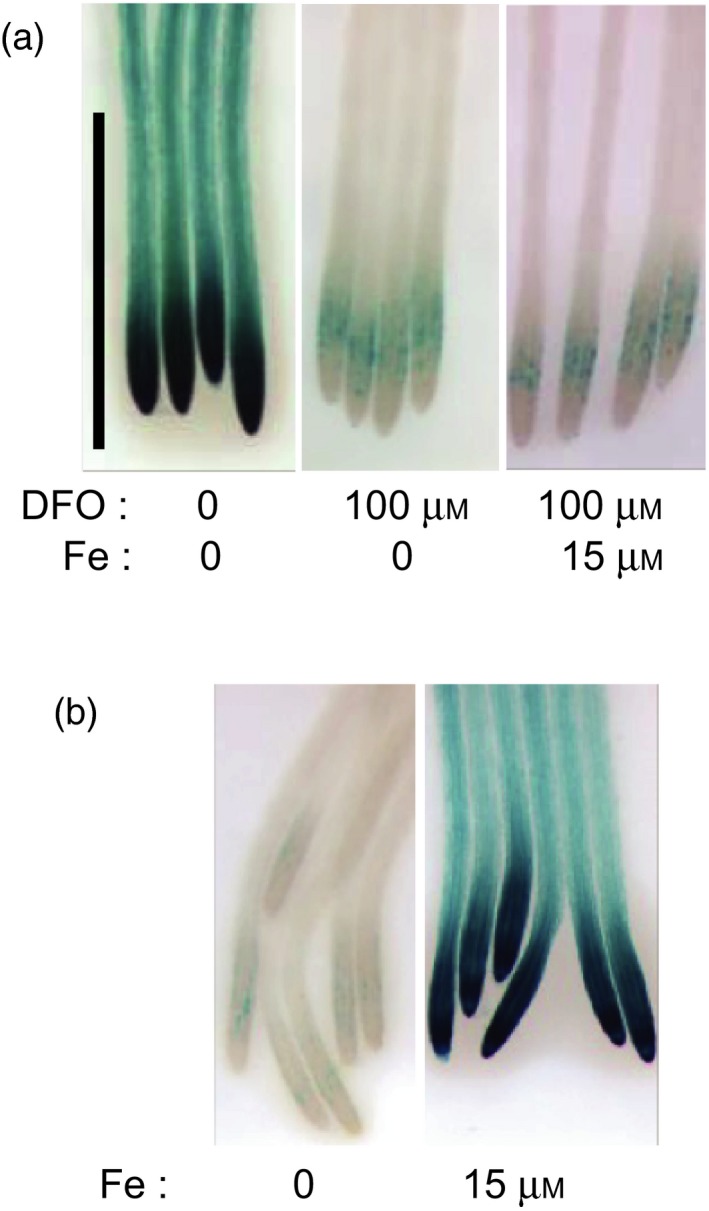
Iron is necessary for the expression of *ALMT1*. (a) Deferoxamine (DFO) inhibits the expression of *pALMT1::GUS*. (b) In a growth medium made with a washed DFO‐treated agar, Fe supplementation restores the expression of *pALMT1::GUS*. Seedlings were grown for 4 days on a pH 5.5 medium not supplemented with phosphate, and supplemented, or not, with the indicated concentrations of DFO and Fe, before GUS staining. Bar = 1 mm.

We observed that even without supplementing the –Pi growth medium with Fe, at pH 5.5 there was still strong expression of *ALMT1* (Figure 2a, left). This prompted us to assay the putative presence of Fe in the agar using inductively coupled plasma–atomic emission spectroscopy (ICP‐AES). This analysis revealed the presence of 38 μg Fe g^−1^ agar (5.5 μm Fe in the final growth medium) (Table S1).

To further test the hypothesis that, in –Pi conditions, the Fe contained in the agar powder is the trigger for *ALMT1* expression, we reduced the availability of Fe in the growth medium using a siderophore. Supplementing the medium with 100 μm deferoxamine (DFO), a potent Fe chelator, strongly diminished the expression of *ALMT1* (Figure 2a). Deferoxamine was effective in chelating Fe, since the addition of 15 μm Fe did not stimulate expression of *ALMT1* (Figure 2a). To avoid the potential toxicity of a high concentration of DFO that could interfere with gene expression, an alternative approach was used to reduce bioavailable Fe from the agar. The agar powder was mixed with DFO and then washed (see Experimental Procedures). We then tested whether the level of expression of *ALMT1* remained low. In –Pi plates made with this washed, DFO‐treated agar, expression of *ALMT1* is almost fully abolished and the addition of 15 μm Fe restored a high level of expression (Figure 2b). We conclude that a strong chelator of Fe suppresses the expression of *ALMT1*. Altogether, these results show that the stimulation of *ALMT1* expression in –Pi conditions is triggered by Fe but not by the Pi deficiency *per se*.

This result prompted us to compare the expression of *ALMT1* with that of *SPX1* (SYG1/Pho81/XPR1), a classical marker of –Pi stress. *SPX1* encodes a protein regulating the activity of the transcription factor PHR1 – a master regulator of –Pi stress which is frequently used as a marker of the –Pi transcriptional response. To monitor its expression, we used WT seedlings containing the *pSPX1::GUS* marker (Duan *et al*., 2008). The *pALMT1::GUS* and *pSPX1::GUS* seedlings were grown in +Pi or –Pi conditions at pH 5.5 or 6.7, and with two different agars or an agarose; the nutrient solution was not supplemented with Fe in any of these media.

**Figure 3:**
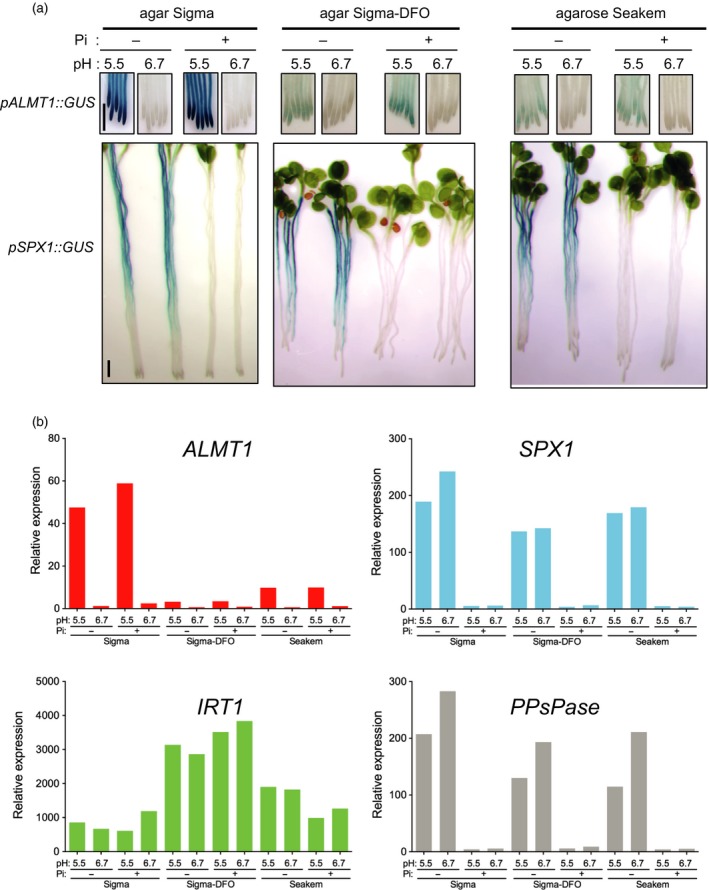
Iron, but not the phosphate‐depleted (–Pi) condition *per se*, stimulates the expression of *pALMT1::GUS*. (a) GUS staining in the primary root of *pALMT1::GUS* and *pSPX1::GUS* seedlings. (b) Expression [by quantitative (q)RT‐PCR] of *ALMT1*,* SPX1*,* IRT1* and *PPsPase* in seedling roots (mean of three technical replicates). Seedlings were grown for 5 days (for the GUS staining experiment) or 3.5 days (for the qRT‐PCR experiment) on a medium not supplemented with Pi or Fe, at pH 6.7 or 5.5. Bars = 1 mm.

As already shown (Balzergue *et al*., 2017), on an agar containing Fe, the expression of *ALMT1* is induced at pH 5.5 but not at pH 6.7, in both –Pi and +Pi conditions (Figure 3a). The stimulation of this expression is much less in media that contain a very low amount of Fe (DFO‐treated agar or on the agarose ‘Seakem'; Figure 3a and Table S1). By contrast, the expression of *SPX1* is induced only in –Pi conditions, whatever the pH or the gelling agent used to prepare the growth medium (Figure 3a). All these observations were confirmed by quantitative reverse transcriptase‐polymerase chain reaction (qRT‐PCR) (Figures 3b and S2), and with the pyrophosphatase [PPi]‐specific phosphatase1 (*PPsPase1*) gene as an additional marker whose expression is highly stimulated by Pi deficiency (Hanchi *et al*., 2018) (Figure 3b).

We also monitored the expression of the *IRON‐REGULATED TRANSPORTER 1* (*IRT1*) gene, a well‐known indicator of Fe deficiency (Vert *et al*., 2002). This marker indicates that, as expected, when grown on the Sigma DFO agar plates seedlings are more Fe starved than on the other plates (Figure 3b). On plates made with the Seakem agar, which contains very low amount of Fe, *IRT1* is also more expressed than with the DFO agar (Figure 3b). These analyses of genes expression further show that *ALMT1* is not stimulated by –Pi *per se*, and strongly suggest that the signal stimulating the expression of *ALMT1* is distinct from that of the SPX1 pathway. By contrast to *ALMT1* mRNA, Al, Fe and pH do not modulate the level of accumulation of *STOP1* mRNA (Figure S3).

### Iron stimulates the accumulation of STOP1 in the nucleus

Using the *pSTOP1::GFP‐STOP1* reporter we previously showed that STOP1 accumulates in the nuclei at the primary root tips of seedlings grown in –Pi (Balzergue *et al*., 2017; Wang *et al*., 2019). Our gene expression analysis of *ALMT1* led us to test whether Fe is involved in this nuclear accumulation of STOP1.

**Figure 4:**
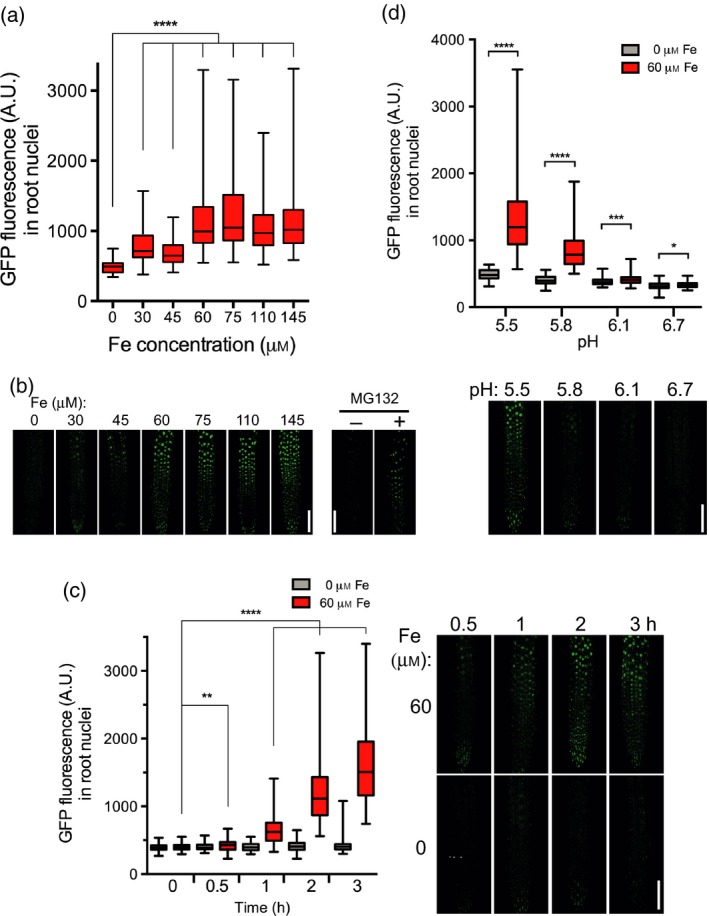
Iron promotes the accumulation of GFP‐STOP1 in root nuclei.The GFP fluorescence was measured (a.u.) in nuclei at the root tip of *pSTOP1::GFP‐STOP1* seedlings. (a) Top: 3‐day‐old seedlings were transferred for 2 h to phosphate‐depleted (–Pi) plates with the indicated concentration of Fe. Bottom: representative pictures used for measurements. (b) MG132 promotes the accumulation of GFP‐STOP1 in nuclei of the root tip. Seedlings carrying the *pSTOP1::GFP‐STOP1* reporter were grown for 3 days in agar (Sigma agar A1296; Table S1) under –Pi without the addition of Fe or Al. Then, they were treated for 4 h with or without 250 μm MG132 before being photographed with a confocal microscope. One representative picture is shown for each condition (see Figure S6 for additional pictures). Note that there is slight accumulation of STOP1 in the nucleus of the untreated control. (c) Left: 3‐day‐old seedlings were transferred for the indicated time to –Pi plates containing 0 or 60 μm Fe. Right: representative pictures used for measurements. (d) Top: 3‐day‐old seedlings were transferred for 2 h to –Pi plates buffered at the indicated pH, containing 0 or 60 μm Fe. Bottom: representative pictures used for measurements. Box plots indicate the median, the 25th to 75th percentiles (box edges) and the minimum to maximum range (whiskers). Mann–Whitney test: ****P < 0.0001; ***P < 0.001; *P < 0.5; NS, not significant (*P* > 0.05); number of nuclei per condition: (a) 94–154; (b) 99–119; (c) 87–121. Bars = 100 μm.

Seedlings carrying the *pSTOP1::GFP‐STOP1* marker were grown for 3 days in non‐inducible conditions (i.e. Seakem agarose without the addition of Pi and Fe that does not stimulate the expression of *ALMT1*, as shown in Figure 3a) then transferred in the same medium supplemented or not with Fe before examining the GFP fluorescence. We first assessed the range of Fe concentrations that stimulate nuclear accumulation of STOP1 in acidic condition (pH 5.5). Figure 4(a) (and Figure S4a for an independent experiment) shows that from 0 to 60 μm Fe GFP fluorescence in the nuclei increases with Fe concentration; a plateau is reached around 60 μm Fe. Note that without Fe we observe small fluorescent dots in cells (Figure 4a bottom, first picture on the left). These dots are autofluorescence of root cell components (i.e. not GFP fluorescence) since a WT seedling that does not carry the GFP marker displays similar dots (Figure S5).

Since we do not detect GFP fluorescence in the cytoplasm in non‐inducible conditions, we hypothesized that the accumulation of GFP‐STOP1 protein in the nucleus results from reduced proteasomal degradation (i.e. instead of translocation from the cytosol). We then tested the effect of MG132, an inhibitor of the 26S proteasome, on the accumulation of GFP‐STOP1 in seedlings grown in a moderate amount of Fe (Sigma agar without added Fe; see Table S1). When treated with MG132, the GFP fluorescence substantially accumulated in the nucleus (Figure 4b; see also Figure S6 for additional pictures of two independent experiments). This result therefore supports the idea that Fe somehow inhibits the degradation of STOP1 by the 26S proteasome.

We then performed a kinetic analysis, at pH 5.5, of the nuclear accumulation of STOP1. Figure 4(c) (and Figure S4b) shows that in seedlings transferred in a medium without Fe added (–Fe), the level of nuclear GFP fluorescence remains low through the 3 h of the analysis. By contrast, after transfer of seedlings onto plates containing 60 μm Fe, the GFP fluorescence in nuclei increases after 30 min and becomes higher than in the –Fe control after 1 h; the fluorescence further increases with time. This confocal analysis, performed with –Pi for all conditions, indicates that Fe, and not the –Pi condition *per se*, rapidly promotes the accumulation of STOP1 in the nucleus.

We have shown previously that the acidity of the growth medium is a crucial parameter for stimulating *ALMT1* expression (Balzergue *et al*., 2017). We therefore tested whether low pH on its own also promotes the nuclear accumulation of STOP1. Figure 4(d) (and Figure S4c) shows that in the presence of 60 μm Fe the level of nuclear GFP fluorescence of GFP‐STOP1 is higher in acidic conditions, whereas at pH 6.7 it is low and does not significantly differ from the –Fe control. This confirms the result shown in Figure 4(a): an acidic pH (below 6.1) without Fe does not stimulate the accumulation of GFP‐STOP1 in the nucleus. These measures show that a low pH is required, but not sufficient, to stimulate nuclear accumulation of STOP1; the accumulation occurs only in acidic conditions supplemented with Fe. This confirms the results shown in Figure 3 on the expression of *ALMT1*.

### Aluminum triggers the accumulation of STOP1 in the nucleus

**Figure 5:**
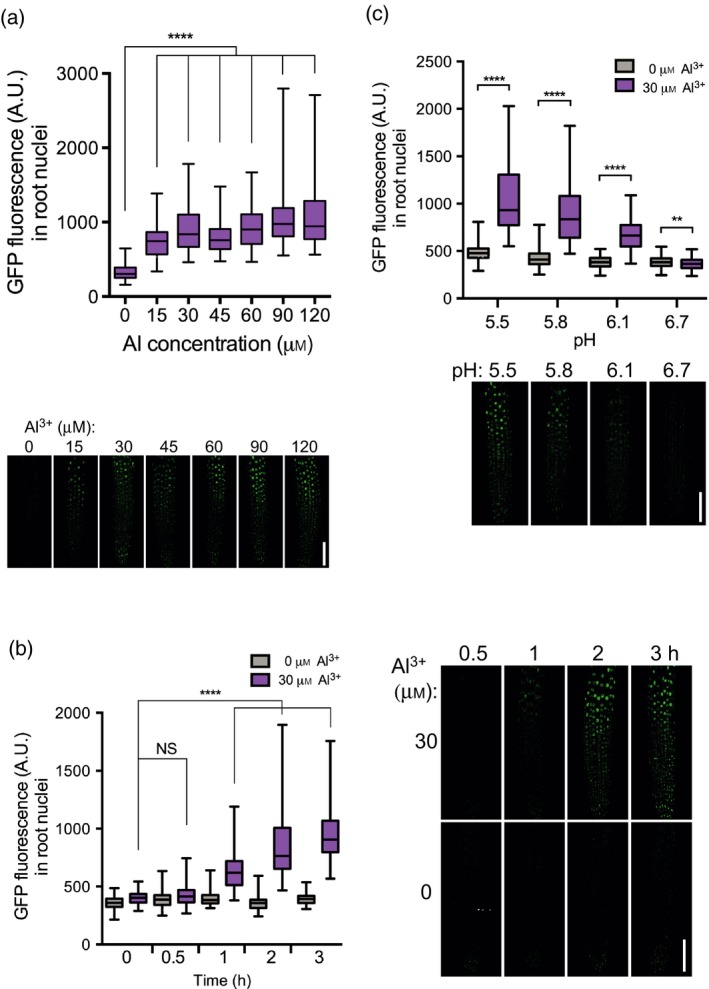
Al^3+^ promotes the accumulation of GFP‐STOP1 in root nuclei.The GFP fluorescence was measured (a.u.) in nuclei at the root tip of *pSTOP1::GFP‐STOP1* seedlings. (a) Top: 3‐day‐old seedlings were transferred for 2 h to phosphate‐depleted (–Pi) plates with the indicated concentration of Al^3+^. Bottom: representative pictures used for measurements. (b) Left: 3‐day‐old seedlings were transferred for the indicated time to –Pi plates containing 0 or 30 μm Al^3+^. Right: representative pictures used for measurements. (c) Top: 3‐day‐old seedlings were transferred for 2 h to –Pi plates buffered at the indicated pH, containing 0 or 30 μm Al^3+^. Bottom: representative pictures used for measurements. Box plots indicate the median, the 25th to 75th percentiles (box edges) and the minimum to maximum range (whiskers). Mann–Whitney test: ****P < 0.0001; **P < 0.01; NS, not significant (*P* > 0.05); number of nuclei per condition: (a) 80–143; (b) 102–125; (c) 86–121. Bars = 100 μm.

STOP1 and ALMT1 participate in resistance against Al^3+^ toxicity, and Al^3+^ stimulates the expression of *ALMT1*. As for Fe (Figure 2b), in seedlings grown on plates made with washed, DFO‐treated agar, expression of *ALMT1* is restored by the addition of 15 μm Al^3+^ (Figure S7). We therefore asked whether Al^3+^ also stimulates the accumulation of STOP1 in the nucleus. Seedlings were grown for 3 days in Seakem agarose plates, and transferred for 2 h to plates supplemented, or not, with Al^3+^ before observing the GFP fluorescence of GFP‐STOP1. Preliminary observations showed GFP fluorescence in the nucleus after transfer to the Al plates. A dose–response curve indicated that 15 μm Al^3+^ is sufficient to detect an accumulation of GFP in the nucleus, and a plateau is reached at about 30 μm Al^3+^ (Figure 5a; see Figure S8a for an independent experiment). A kinetic analysis indicates that 1 h after transfer the GFP signal is already significantly higher than the control not supplemented with Al (Figures 5b and S8b). The signal increases further through the 3 h of the analysis.

As for Fe, a low pH (from 5.5 to 6.7) is required for Al to promote the nuclear accumulation of GFP‐STOP1 (Figures 5c and S8c). These experiments show that Al, like Fe, rapidly triggers nuclear accumulation of STOP1 when the growth conditions are acidic.

### The overaccumulation of STOP1 in root nuclei of *als3* is dependent on Fe

**Figure 6:**
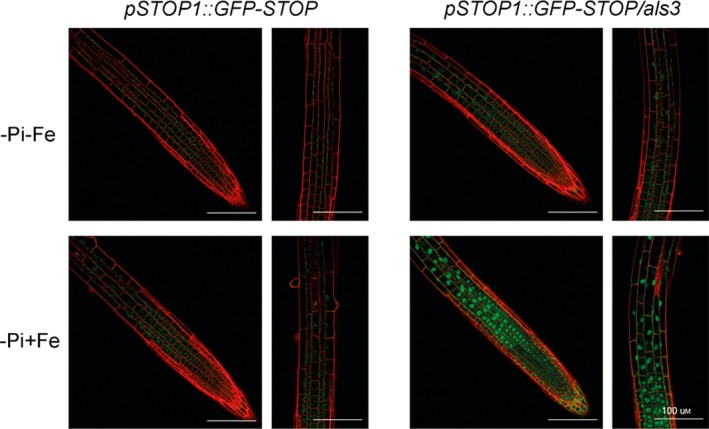
ALS3 represses STOP1 accumulation in root nuclei. Wild‐type (WT) and *als3* mutant seedlings carrying the *pSTOP1::GFP‐STOP1* construct were grown for 3 days on a phosphate‐ and Fe‐depleted (–Pi–Fe) plate (made with Seakem agar), transferred to –Pi–Fe or –Pi + 60 μm Fe plates for 2 h, and GFP fluorescence was visualized by confocal microscopy. To avoid saturated GFP fluorescence in the images, the microscope was set on the highest fluorescence (as detected in *als3*). Bars = 100 μm.

Using the *pSTOP1::GFP‐STOP1* marker we have previously shown that in the *als3* and *star1* mutants, the accumulation of GFP‐STOP1 in root nuclei is much higher than in the WT (Wang *et al*., 2019). We thus asked whether this overaccumulation depends on Fe. We confirmed that, when grown on Seakem agarose plates supplemented with 60 μm Fe, the *als3* mutant accumulates much more GFP‐STOP1 in nuclei than the WT control (Figure 6). In –Fe, this accumulation is suppressed in both the WT and the *als3* mutant (Figure 6). This result was confirmed with the DFO agar (Figure S9). Therefore, the enhanced accumulation of nuclear GFP‐STOP1 in *als3* root nuclei depends on Fe. Our results show that Fe is critical for STOP1 to accumulate in nucleus, and ALS3 represses this accumulation in an Fe‐dependent manner.

## Discussion

We, and others, have previously shown that several aspects of the root growth arrest under –Pi conditions are dependent on Fe (Svistoonoff *et al*., 2007; Ward *et al*., 2008; Muller *et al*., 2015; Dong *et al*., 2017; Gutiérrez‐Alanís *et al*., 2017; Singh *et al*., 2018; Wang *et al*., 2019), but the exact relationships between –Pi and Fe were unclear. Here, we focused our work on STOP1 signaling and succeeded in dissociating the effect of –Pi itself from the role of Fe in STOP1 signaling.

We compared the expression of *SPX1* and *PPsPase* genes as markers of systemic –Pi stress with that of *ALMT1* that concerns local –Pi stress. We show that, in the root, Fe stimulates the expression of *ALMT1* regardless of the concentration of Pi and that –Pi–Fe as well as +Pi–Fe conditions decrease the expression of *ALMT1*. In addition, we demonstrate that the accumulation of STOP1 in root tip nuclei is dependent on Fe. By contrast, the expression of *SPX1* and *PPsPase* only occurs under –Pi and it is not correlated with the availability of Fe in the medium (Figures 3 and S2). Supporting this conclusion, our previous results showed that the expression of *ALMT1* in –Pi does not depend on *PHR1* and *PHL1* genes (Balzergue *et al*., 2017), two targets of SPX1 regulation (Puga *et al*., 2014).

We thus demonstrate that, under –Pi conditions, it is the Fe and not the –Pi condition itself that stimulates the accumulation of STOP1 in root nuclei and the transcription of *ALMT1*. Under +Pi, Fe is probably associated with Pi molecules, thus hampering the Fe‐dependent regulation of nuclear STOP1 and the catalysis of ROS production, whereas, under –Pi, Fe is more available to stimulate these two processes.

Therefore, the STOP1 signaling pathway is clearly distinct from the systemic –Pi signaling, at least the PHR1‐ and PHL1‐dependent pathways.

### Iron and Al stimulate the accumulation of STOP1 in the nucleus

Our work demonstrates that Fe and Al^3+^ stimulate the accumulation of STOP1 in the nucleus. In previous works by others, the cellular localization of Arabidopsis STOP1 and STOP1 homologs was assessed by transient expression in onion cells, Arabidopsis protoplasts, protoplasts derived from rice callus, tobacco leaves or *Nicotiana benthamiana* leaves. In all these assays, the STOP1 proteins, fused to the GFP, were localized in the nucleus (Sawaki *et al*., 2009, 2014; Yamaji *et al*., 2009; Fan *et al*., 2015; Wang *et al*., 2017; Che *et al*., 2018; Daspute *et al*., 2018; Huang *et al*., 2018; Wu *et al*., 2018). In these assays, the growth media used were not Pi deficient or particularly enriched in Fe or Al^3+^. This suggests that, in these experimental conditions, the pathway stimulating or repressing the accumulation of STOP1 in the nucleus is constitutively active or inactive, respectively. Supporting this last idea, an immunostaining experiment showed a constitutive nuclear localization of OsART1 in WT root cells (i.e. roots not affected by Al treatment) (Yamaji *et al*., 2009). Another possibility is that overexpression of the STOP1 protein saturates the putative regulatory mechanism governing its nuclear accumulation. Alternatively, these STOP1 proteins are differently regulated in these cells compared with the root cells in Arabidopsis.

We have previously shown that under neutral pH conditions root growth is not arrested in –Pi (Svistoonoff *et al*., 2007) and *ALMT1* is not expressed (Balzergue *et al*., 2017). These two phenomena are now explained (at least partially) as we show that under low pH without Fe or Al^3+^, STOP1 does not accumulate in the nucleus and, reciprocally, in the presence of Fe or Al^3+^, but at pH 6.7, STOP1 does not accumulate in the nucleus either (Figures 4 and 5). The low pH is thus a necessary but not sufficient condition for stimulating nuclear STOP1.

Our qRT‐PCR result shows that *IRT1* – a gene whose expression is induced when Fe is poorly available, as on DFO agar (Figure 3b) – is not (or is only poorly) induced at pH 6.7 (Figure 3b; Sigma agar and Seakem agarose), indicating that seedlings are not Fe‐deficient. Nevertheless, this pH prevents the accumulation of STOP1 in the nucleus. This suggests that different Fe signaling or different pools or forms of Fe regulate *IRT1* expression and nuclear accumulation of STOP1. Knowing that Fe^2/3+^ and Al^3+^ are only soluble under low pH, regulation of STOP1 seems sensitive to the cationic form of Fe and Al.

The first *stop1* mutant was originally identified for its defective root growth on agar plates made with a MS medium at pH 4.3 (Iuchi *et al*., 2007). The authors confirmed the hypersensitivity to low pH by using hydroponic culture; this growth medium contained only submicromolar concentrations of Fe (and no Pi). According to the results presented here, under acidic conditions without Fe (or Al^3+^), STOP1 is poorly accumulated in the nucleus. Then, if STOP1 does not accumulate in nucleus under these growth conditions, how does it participate in resistance against low pH? One hypothesis is that at pH 4.3 a very low amount of Fe is sufficient to stimulate the accumulation of STOP1 for triggering the expression of genes for H^+^ tolerance. Alternatively, STOP1 might have already started promoting the expression of genes involved in H^+^ tolerance before (i.e. during embryogenesis) seedlings encounter acidic conditions. A transcriptomic study of seedlings grown in an acidic environment showed that the *stop1* mutant is defective for the expression of several genes, including genes involved in cell wall composition or modeling (Sawaki *et al*., 2009). Furthermore, physiological studies suggested that *stop1* seedlings are defective in a mechanism alleviating H^+^ toxicity, perhaps via Ca^2+^ stabilization of the cell wall (Kobayashi *et al*., 2013b). It is therefore tempting to speculate that even during WT embryogenesis STOP1 is participating in the making of root cells with a cell wall able to tolerate H^+^ a few days after germination, and in *stop1* seeds the root cell walls are somehow altered, leading to reduced root growth under acidic conditions.

### ALS3 and STAR1 repress the accumulation of STOP1 in the nucleus

In a previous work, Wang *et al*. demonstrated that, together, ALS3 and STAR1 repress the accumulation of STOP1 in the nucleus (Wang *et al*., 2019). ALS3 and STAR1 associate to form an ABC‐type transporter located in the tonoplast. We thus inferred a model whereby a cytosolic metabolite stimulates the accumulation of STOP1 in the nucleus, and that ALS3–STAR1 transporter pumps this metabolite from the cytosol to the vacuole. According to this model, the cytosolic concentration of this metabolite is higher in *als3* and *star1* mutants than in the WT, thereby increasing nuclear STOP1.

We showed here that overaccumulation of STOP1 in the nucleus of *als3* mutant is abrogated when the seedling grows in an Fe‐depleted medium (Figure 6). Since our results on nuclear STOP1 with Al^3+^ are similar to those with Fe, this metabolite could be the trivalent metal (Fe^3+^ or Al^3+^) or a Fe‐ (or Al^3+^)‐containing molecule. This hypothesis would fit with the role of ALS3–STAR1 in Al^3+^ tolerance. However, we cannot exclude that this metabolite does not contain Fe (or Al^3+^).

The *STOP1*,* ALS3* and *STAR1* genes are all expressed in the root tip (Larsen *et al*., 2005; Balzergue *et al*., 2017; Dong *et al*., 2017; Mora‐Macias *et al*., 2017); this is consistent with their role as a shared functional unit.

### The two effects of Fe on root growth arrest

A two‐branched regulatory pathway modulates root growth arrest: the STOP1/ALMT1/ALS3/STAR1 branch and the LPR1/PDR2 branch (Abel, 2017; Balzergue *et al*., 2017; Wang *et al*., 2019). These two branches converge on the Fe‐dependent production of ROS in the cell wall. In the *lpr1* mutant the expression of *ALMT1* is not altered compared with the WT (Balzergue *et al*., 2017). This means that, in the *lpr1* mutant, STOP1 is as active as in the WT. Since the *lpr1* root tip accumulates far less extracellular Fe and ROS than the WT, ROS and extracellular Fe do not seem to be crucial for activating STOP1 for *ALMT1* expression. Instead, our present work with ALS3 indicates that an intracellular metabolite (containing Fe or Al^3+^?) activates the STOP1 branch.

**Figure 7:**
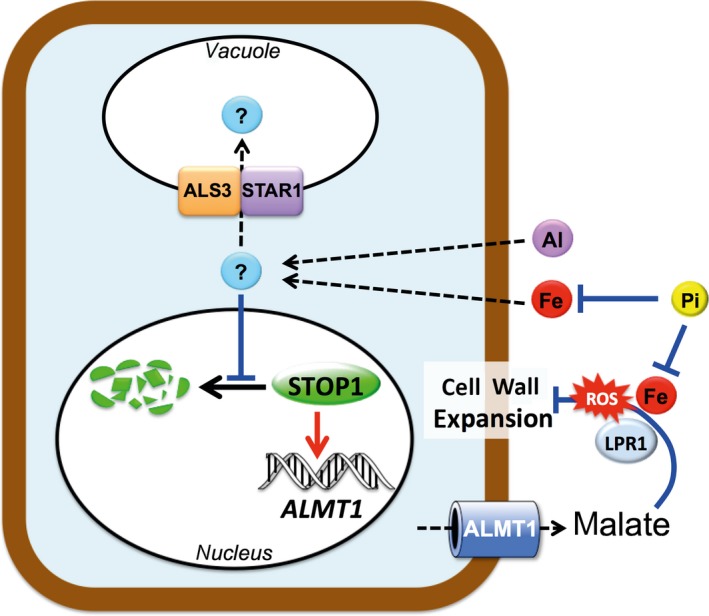
Model depicting the roles of Fe and Al in STOP1 signaling and root growth in relation to phosphate (Pi) availability. Phosphate reversibly inactivates Fe and Al by forming a complex with them. Under –Pi conditions and low pH, Fe, Al or another unknown molecule accumulates in the cell where it decreases proteasomal degradation of STOP1, thereby stimulating the accumulation of STOP1 in the nucleus; nuclear STOP1 activates the transcription of *ALMT1*. The tonoplast‐anchored ALS3 and STAR1 proteins pump the Fe, Al or unknown molecule from the cytosol to the vacuole compartment, thereby decreasing its concentration in the cytosol. This reduces the accumulation of STOP1 in the nucleus and therefore the transcription of *ALMT1*. The ALMT1 transporter exudes malate in the apoplast where, together with Fe and the ferroxidase LPR1, they generate reactive oxygen species (ROS) that inhibit cell wall expansion. Exuded malate also chelates Al^3+^, therefore preventing its toxicity (not presented in the scheme). Red arrows, activation; black arrow, proteasomal degradation; blue blunt arrows, repression; dashed arrows, transfer between compartments.

Figure 7 shows a model summarizing our current knowledge of this two‐branched pathway. The Pi ions inactivate Fe by forming a complex with it. Under –Pi conditions, Fe is released from this complex. Iron – or an unknown compound – accumulates in the cell where it stimulates the accumulation of STOP1 in the nucleus by inhibiting its proteasomal degradation. Inside the nucleus, STOP1 activates the transcription of *ALMT1*. The ALMT1 protein exudes malate in the apoplast where, together with Fe and the ferroxidase LPR1, they generate ROS that inhibit cell wall expansion via the cross‐linking activity of cell wall peroxidases. In the tonoplastic membrane, ALS3 and STAR1 pump Fe – or the unknown compound – from the cytosol to the vacuole compartment. It follows that the concentration of Fe, or the unknown compound, in the cytosol decreases and this reduces the accumulation of STOP1 in the nucleus, and therefore also reduces the expression of *ALMT1*.

Note that Fe has two effects: it directly or indirectly activates the accumulation of STOP1 in the nucleus and it also participates in the generation of ROS in the apoplast. These two effects can be uncoupled; for example, the *lpr1* mutant still expresses *ALMT1* but its root growth is not inhibited under –Pi.

For Al, the model only partially applies because the exuded malate prevents the inhibitory effect of toxic Al^3+^ on root growth. In addition, we do not know whether ALS3 and STAR1 pump Al^3+^. In any case, Fe and Al^3+^ share several characteristics in the nuclear accumulation of STOP1: dependence on low‐pH, similar kinetics and the negative role of ALS3–ASTAR1. This could mean that Fe and Al^3+^ have some common signaling steps activating STOP1. Many points remain obscure or are not yet demonstrated in this model – in particular, how cellular Fe (and Al^3+^) regulates STOP1.

### How do Fe and Al stimulate the accumulation of STOP1 in the nucleus?

Few Fe‐sensing proteins have been identified in plants (Kobayashi and Nishizawa, 2012). The HRZ [hemerythrin motif‐containing really interesting new gene (RING)‐and zinc‐finger] proteins BRUTUS (BTS), BTS‐like, OsHRZ1 and OsHRZ2 are E3 ligases involved in the low‐Fe response (Long *et al*., 2010; Kobayashi *et al*., 2013a; Hindt *et al*., 2017). They target to proteasomal degradation several basic helix–loop–helix transcription factors such as POPEYE (PYE) and PYE‐like factors such as bHLH104 and bHLH105, which are positive regulators of the low‐Fe response. Under Fe‐sufficient conditions, the binding of Fe to the hemerythrin domains destabilizes the HRZ proteins, thereby relieving the transcriptional response to low Fe (Kobayashi *et al*., 2013a; Selote *et al*., 2015). Also involved in the transcriptional regulation of Fe homeostasis are the IDEF1 and IDEF2 proteins that bind Fe, as well as Zn (Kobayashi *et al*., 2012).

Here, we have shown that MG132, an inhibitor of the 26S proteasome, substantially increases the level of GFP‐STOP1 accumulated in the nucleus in seedlings grown in poorly inductive conditions (i.e. low Fe; Figures 4b and S6). This indicates that under poorly or non‐inductive conditions, STOP1 is degraded by the 26S proteasome pathway. Recently, Zhang *et al*. (2019) found an F‐box protein, RAE1, that promoted the degradation of STOP1 via the 26S‐proteasome pathway. The REA1 protein interacts with and triggers the 26S proteasome‐dependent degradation of STOP1. Compared with the WT, seedlings homozygous for the loss‐of‐function *rae1* mutation accumulate more STOP1 proteins and *ALMT1* mRNAs. When grown under –Pi conditions, the *rae1* seedlings have a primary root that is shorter than that of the WT, and when grown with toxic amounts of Al it is longer. RAE1 is therefore a strong candidate mediating STOP1 degradation in our Fe‐ and Al‐depleted growth conditions.

The RAE1 and STOP1 proteins do not contain an obvious Fe‐ or Al‐binding domain. How Al^3+^ is sensed in plants is not known (Kochian *et al*., 2015) and thus the activation of STOP1 by Al^3+^ remains an open question. But, since both Fe and Al^3+^ stimulate the accumulation of STOP1 in the nucleus with similar kinetics, in the same root cells and only under acidic conditions, it is tempting to speculate that these two cations share a sensing mechanism. Further work will be needed to understand this mechanism.

## Conclusion

In acidic soils, toxic Al^3+^ and Fe, in conjunction with Pi deficiency limit crop growth (Kochian *et al*., 2004). There is increasing evidence that, at the cellular level, toxic Al^3+^ shares some common targets and processes with the effects of Fe cations in acidic and low‐Pi conditions (Abel, 2017). We now demonstrate that Al^3+^ and Fe also share at least one signaling step: the accumulation of STOP1 in the nucleus. Our study therefore contributes to further dissection of the sensing and signaling pathways of Al and Fe in plants.

## Experimental Procedures

### Plant material

The Arabidopsis WT (Col^*er105*^), *pALMT1::GUS*_*#2*_, *pSTOP1::GFP‐STOP1*_*#B10*_ and *als3*;* pSTOP1::GFP‐STOP1*_*#B10*_ lines were described previously (Balzergue *et al*., 2017; Wang *et al*., 2019); *pSPX1::GUS* is in the Col‐0 background (Duan *et al*., 2008).

### Seedling growth

Seeds were surface‐sterilized for 5 min in a solution containing 70% ethanol and 0.05% sodium dodecyl sulfate, and washed twice with 96% ethanol. The nutrient solution was the same as used previously (Balzergue *et al*., 2017). The agar (8 g L^−1^) and agarose (7 g L^−1^) for plates were from Sigma‐Aldrich (A1296 #BCBL6182V, https://www.sigmaaldrich.com/) and Lonza (Seakem LE agarose, https://www.lonza.com/), respectively. The media were buffered at pHs ranging from 5.5 to 6.7 with 3.4 mm 2‐(*N*‐morpholino)ethanesulfonic acid and at pH 7.1 with 3 mM 1,4‐piperazinediethanesulfonic acid disodium salt. The different plates were prepared by mixing the melted autoclaved 1× agar or agarose medium (composition as above) with the buffer. Solutions of AlCl_3_ and FeCl_2_ were filtrated before adding to the plates.

For GFP fluorescence experiments, five seeds were sown on a piece of sterile nylon square (1 cm × 1 cm, mesh size 10 μm), itself lying on the agar plate. After 3 days of growth on the agar plate, the nylon meshes carrying the seedlings were transferred to the new plates containing, or not, different concentrations of Al or Fe.

For the experiments with MG132, nylon meshes carrying the 3‐day‐old seedlings were transferred on a 10‐μl droplet (liquid extracted from Sigma‐Aldrich agar A1296 #BCBL6182V from which the seedlings were pre‐grown) containing, or not, different concentrations of MG132. In all experiments, the pH of the growth medium for germination and for the subsequent transfer was identical.

For the qRT‐PCR experiment, seeds were sown side by side in a line, on a 5‐cm strip of nylon meshes. Three to 3.5 days after sowing the whole roots of seedlings were harvested and mRNA extracted.

### Agar treatment with DFO

We used two methods to reduce bioavailable Fe in the growth medium. For the DFO agar, 100 μm of DFO was added to the 1× melted autoclaved growth medium just before pouring the plates. For the washed DFO‐treated agar, we prepared the agar as follows: 4 g of Sigma‐Aldrich agar (A1296 #BCBL6182V) was added to 100 ml of MilliQ water containing 600 μm of DFO and stirred for 15 h. The agar was then washed by centrifugation (520 ***g***) through a nylon mesh (mesh size 10 μm); this washing step with MilliQ water was carried out twice. This washed DFO agar was then used to prepare the growth plates.

### Quantification of Al, Fe and P in the agars and agarose

Dried samples of agars and agarose were mineralized by the addition of 300 μl of 70% HNO_3_ (ICP grade, JT Baker https://www.fishersci.fr/) by incubation overnight in an oven at 80°C. The solutions were diluted to a final volume of 5 ml by the addition of distilled water. The Al, Fe and P were quantified using ICP‐AES (5110 SVDV, Agilent Technologies, https://www.agilent.com/). The concentrations of metals were determined using standard curves obtained from solutions made with ICP‐grade elements.

### Quantitative RT‐PCR

Total RNA was extracted from whole roots using the Direct‐Zol™ RNA MiniPrep (Zymo Research, https://www.zymoresearch.com/) and treated with the RNase‐free DNase Set (Zymo Research) according to the manufacturer's instructions. Reverse transcription was performed on 500 ng of total RNA using qScript™ cDNA SuperMix (Quantabio, https://www.quantabio.com/). Quantitative PCR was performed on a 480 LightCycler thermocycler (Roche, https://www.roche.com/) using the manufacturer's instructions with Light Cycler 480 SYBR Green I Master (Roche) and the primers listed in Table S2. We used the Tubulin gene (*At5g62690*) as a reference gene for normalization and the quantification of gene expression was as according to published method (Pfaffi, 2001).

### GUS histochemical staining

The GUS staining of Arabidopsis seedlings was conducted as previously described (Balzergue *et al*., 2017), except that the seedlings were incubated for 40 min in the GUS staining solution.

### Quantification of GFP fluorescence

Images were collected on a Zeiss LSM780 confocal microscope (Carl Zeiss, https://www.zeiss.com/) using a ×20 dry objective (Plan Apo NA 0.80). The GFP was excited with an Ar ion laser (488 nm). Emitted light was collected from 493 to 538 nm for GFP, using the MBS 488 filter. All nuclei were imaged using the same conditions of gain, offset and resolution and with a pinhole setting of 1 a.u. The quantification of GFP fluorescence was carried out as follows: a *Z*‐stack of nine images (separated by a distance of 1.8 μm) imaged representative nuclei on the surface of the root. Images were acquired in 12 bits using Zen black software (SP2 v.11.0, 2012, Carl Zeiss), then converted to a maximum projection image. The average nuclear fluorescence intensities were quantified using Zen blue software (Zen 2 blue edition, v.2.0.0.0, Carl Zeiss), by drawing identical regions of interest. A 120 μm × 60 μm rectangle was defined at 200 μm from a root tip (roughly in the transition zone), in which a region of interest (6 μm × 6 μm) inside each nucleus was defined for the measurement of fluorescence.

## Conflict of interest

The authors declare no competing interests.

## Supporting information


**Figure S1.** Under neutral conditions, *ALMT1* is not expressed.

**Figure S2.** Expression of *ALMT1*,* SPX1* and *PPsPase* in seedlings roots.

**Figure S3.** Analysis of *STOP1* mRNA expression.

**Figure S4.** Iron promotes the accumulation of GFP‐STOP1 in root nuclei.

**Figure S5.** Autofluorescence in the root tip of non‐transgenic wild‐type seedlings.

**Figure S6.** The 26S proteasome inhibitor MG132 promotes accumulation of GFP‐STOP1 in root nuclei.

**Figure S7.** Aluminum stimulates the expression of *pALMT1::GUS*.

**Figure S8.** Al^3+^ promotes the accumulation of GFP‐STOP1 in root nuclei.

**Figure S9.** ALS3 represses the accumulation of STOP1 in root nuclei.

**Table S1.** Aluminum, Fe and P content (μg/100 mg agar or agarose).
**Table S2.** Primer sequences.

## References

[tpj14374-bib-0001] Abel, S. (2017) Phosphate scouting by root tips. Curr. Opin. Plant Biol. 39, 168–177. 28527590 10.1016/j.pbi.2017.04.016

[tpj14374-bib-0002] Balzergue, C. , Dartevelle, T. , Godon, C. ***et al*.** (2017) Low phosphate activates STOP1‐ALMT1 to rapidly inhibit root cell elongation. Nat. Commun. 8, 15300. 28504266 10.1038/ncomms15300 PMC5440667

[tpj14374-bib-0003] Bouain, N. , Shahzad, Z. , Rouached, A. ***et al*.** (2014) Phosphate and zinc transport and signalling in plants: toward a better understanding of their homeostasis interaction. J. Exp. Bot. 65, 5725–5741. 25080087 10.1093/jxb/eru314

[tpj14374-bib-0004] Briat, J.F. , Rouached, H. , Tissot, N. , Gaymard, F. and Dubos, C. (2015) Integration of P, S, Fe, and Zn nutrition signals in Arabidopsis thaliana: potential involvement of PHOSPHATE STARVATION RESPONSE 1 (PHR1). Front. Plant Sci. 6, 290. 25972885 10.3389/fpls.2015.00290 PMC4411997

[tpj14374-bib-0005] Che, J. , Tsutsui, T. , Yokosho, K. , Yamaji, N. and Ma, J.F. (2018) Functional characterization of an aluminum (Al)‐inducible transcription factor, ART2, revealed a different pathway for Al tolerance in rice. New Phytol. 220, 209–218. 29888411 10.1111/nph.15252

[tpj14374-bib-0006] Daspute, A.A. , Kobayashi, Y. , Panda, S.K. ***et al*.** (2018) Characterization of CcSTOP1; a C2H2‐type transcription factor regulates Al tolerance gene in pigeonpea. Planta, 247, 201–214. 28921050 10.1007/s00425-017-2777-6

[tpj14374-bib-0007] Dong, J. , Pineros, M.A. , Li, X. ***et al*.** (2017) An Arabidopsis ABC Transporter Mediates Phosphate Deficiency‐Induced Remodeling of Root Architecture by Modulating Iron Homeostasis in Roots. Mol. Plant, 10, 244–259. 27847325 10.1016/j.molp.2016.11.001

[tpj14374-bib-0008] Duan, K. , Yi, K. , Dang, L. , Huang, H. , Wu, W. and Wu, P. (2008) Characterization of a sub‐family of Arabidopsis genes with the SPX domain reveals their diverse functions in plant tolerance to phosphorus starvation. Plant J. 54, 965–975. 18315545 10.1111/j.1365-313X.2008.03460.x

[tpj14374-bib-0009] Fan, W. , Lou, H.Q. , Gong, Y.L. ***et al*.** (2015) Characterization of an inducible C2H2‐type zinc finger transcription factor VuSTOP1 in rice bean (Vigna umbellata) reveals differential regulation between low pH and aluminum tolerance mechanisms. New Phytol. 208, 456–468. 25970766 10.1111/nph.13456

[tpj14374-bib-0010] Gutiérrez‐Alanís, D. , Yong‐Villalobos, L. , Jiménez‐Sandoval, P. ***et al*.** (2017) Phosphate starvation‐dependent iron mobilization induces CLE14 expression to trigger root meristem differentiation through CLV2/PEPR2 signaling. Dev. Cell, 41, 555–570. 28586647 10.1016/j.devcel.2017.05.009

[tpj14374-bib-0011] Hanchi, M. , Thibaud, M.C. , Legeret, B. ***et al*.** (2018) The phosphate fast‐responsive genes PECP1 and PPsPase1 affect phosphocholine and phosphoethanolamine content. Plant Physiol. 176, 2943–2962. 29475899 10.1104/pp.17.01246 PMC5884592

[tpj14374-bib-0012] Hindt, M.N. , Akmakjian, G.Z. , Pivarski, K.L. ***et al*.** (2017) BRUTUS and its paralogs, BTS LIKE1 and BTS LIKE2, encode important negative regulators of the iron deficiency response in Arabidopsis thaliana. Metallomics, 9, 876–890. 28620661 10.1039/c7mt00152e PMC5558852

[tpj14374-bib-0013] Hinsinger, P. (2001) Bioavailability of soil inorganic P in the rhizosphere as affected by root‐induced chemical changes: a review. Plant Soil, 237, 173–195.

[tpj14374-bib-0014] Hirsch, J. , Marin, E. , Floriani, M. ***et al*.** (2006) Phosphate deficiency promotes modification of iron distribution in Arabidopsis plants. Biochimie, 88, 1767–1771. 16757083 10.1016/j.biochi.2006.05.007

[tpj14374-bib-0015] Hoekenga, O.A. , Maron, L.G. , Pineros, M.A. ***et al*.** (2006) AtALMT1, which encodes a malate transporter, is identified as one of several genes critical for aluminum tolerance in Arabidopsis. Proc. Natl Acad. Sci. USA, 103, 9738–9743. 16740662 10.1073/pnas.0602868103 PMC1480476

[tpj14374-bib-0016] Huang, C.F. , Yamaji, N. and Ma, J.F. (2010) Knockout of a bacterial‐type ATP‐binding cassette transporter gene, AtSTAR1, results in increased aluminum sensitivity in Arabidopsis. Plant Physiol. 153, 1669–1677. 20498340 10.1104/pp.110.155028 PMC2923911

[tpj14374-bib-0017] Huang, S. , Gao, J. , You, J. ***et al*.** (2018) Identification of STOP1‐Like proteins associated with aluminum tolerance in sweet sorghum (Sorghum bicolor L.). Front. Plant Sci. 9, 258. 29541086 10.3389/fpls.2018.00258 PMC5835670

[tpj14374-bib-0018] Iuchi, S. , Koyama, H. , Iuchi, A. ***et al*.** (2007) Zinc finger protein STOP1 is critical for proton tolerance in Arabidopsis and coregulates a key gene in aluminum tolerance. Proc. Natl Acad. Sci. USA, 104, 9900–9905. 17535918 10.1073/pnas.0700117104 PMC1887543

[tpj14374-bib-0019] Kobayashi, T. and Nishizawa, N.K. (2012) Iron uptake, translocation, and regulation in higher plants. Annu. Rev. Plant Biol. 63, 131–152. 22404471 10.1146/annurev-arplant-042811-105522

[tpj14374-bib-0020] Kobayashi, T. , Itai, R.N. , Aung, M.S. , Senoura, T. , Nakanishi, H. and Nishizawa, N.K. (2012) The rice transcription factor IDEF1 directly binds to iron and other divalent metals for sensing cellular iron status. Plant J. 69, 81–91. 21880076 10.1111/j.1365-313X.2011.04772.x

[tpj14374-bib-0021] Kobayashi, T. , Nagasaka, S. , Senoura, T. , Itai, R.N. , Nakanishi, H. and Nishizawa, N.K. (2013a) Iron‐binding haemerythrin RING ubiquitin ligases regulate plant iron responses and accumulation. Nat. Commun. 4, 2792. 24253678 10.1038/ncomms3792 PMC3905729

[tpj14374-bib-0022] Kobayashi, Y. , Kobayashi, Y. , Watanabe, T. ***et al*.** (2013b) Molecular and physiological analysis of Al³⁺ and H⁺ rhizotoxicities at moderately acidic conditions. Plant Physiol. 163, 180–192. 23839867 10.1104/pp.113.222893 PMC3762639

[tpj14374-bib-0023] Kochian, L.V. , Hoekenga, O.A. and Pineros, M.A. (2004) How do crop plants tolerate acid soils? Mechanisms of aluminum tolerance and phosphorous efficiency. Annu. Rev. Plant Biol. 55, 459–493. 15377228 10.1146/annurev.arplant.55.031903.141655

[tpj14374-bib-0024] Kochian, L.V. , Pineros, M.A. , Liu, J.P. and Magalhaes, J.V. (2015) Plant adaptation to acid soils: the molecular basis for crop aluminum resistance. Annu. Rev. Plant Biol. 66, 571–598. 25621514 10.1146/annurev-arplant-043014-114822

[tpj14374-bib-0025] Larsen, P.B. , Geisler, M.J. , Jones, C.A. , Williams, K.M. and Cancel, J.D. (2005) ALS3 encodes a phloem‐localized ABC transporter‐like protein that is required for aluminum tolerance in Arabidopsis. Plant J. 41, 353–363. 15659095 10.1111/j.1365-313X.2004.02306.x

[tpj14374-bib-0026] Long, T.A. , Tsukagoshi, H. , Busch, W. , Lahner, B. , Salt, D.E. and Benfey, P.N. (2010) The bHLH transcription factor POPEYE regulates response to iron deficiency in Arabidopsis roots. Plant Cell. 22, 2219–2236. 20675571 10.1105/tpc.110.074096 PMC2929094

[tpj14374-bib-0027] Lynch, J.P.B. and Brown, K.M. (2001) Topsoil foraging ‐ an architectural adaptation of plants to low phosphorus availability. Plant Soil, 237, 225–237.

[tpj14374-bib-0028] Misson, J. , Raghothama, K.G. , Jain, A. ***et al*.** (2005) A genome‐wide transcriptional analysis using Arabidopsis thaliana Affymetrix gene chips determined plant responses to phosphate deprivation. Proc. Natl Acad. Sci. USA, 102, 11934–11939. 16085708 10.1073/pnas.0505266102 PMC1188001

[tpj14374-bib-0029] Mora‐Macias, J. , Ojeda‐Rivera, J.O. , Gutierrez‐Alanis, D. ***et al*.** (2017) Malate‐dependent Fe accumulation is a critical checkpoint in the root developmental response to low phosphate. Proc. Nat. Aca. Sci. USA, 114, E3563–E3572. 10.1073/pnas.1701952114 PMC5410833 28400510

[tpj14374-bib-0030] Muller, J. , Toev, T. , Heisters, M. ***et al*.** (2015) Iron‐dependent callose deposition adjusts root meristem maintenance to phosphate availability. Dev. Cell, 33, 216–230. 25898169 10.1016/j.devcel.2015.02.007

[tpj14374-bib-0031] Pfaffi, M.W. (2001) A new mathematical model for relative quantification in real‐time RT‐PCR. Nucleic Acids Res. 29, e45. 11328886 10.1093/nar/29.9.e45 PMC55695

[tpj14374-bib-0032] Puga, M.I. , Mateos, I. , Charukesi, R. ***et al*.** (2014) SPX1 is a phosphate‐dependent inhibitor of PHOSPHATE STARVATION RESPONSE 1 in Arabidopsis. Proc. Natl Acad. Sci. USA, 111, 14947–14952. 25271326 10.1073/pnas.1404654111 PMC4205628

[tpj14374-bib-0033] Puga, M.I. , Rojas‐Triana, M. , De Lorenzo, L. , Leyva, A. , Rubio, V. and Paz‐Ares, J. (2017) Novel signals in the regulation of Pi starvation responses in plants: facts and promises. Curr. Opin. Plant Biol. 39, 40–49. 28587933 10.1016/j.pbi.2017.05.007

[tpj14374-bib-0034] Reymond, M. , Svistoonoff, S. , Loudet, O. , Nussaume, L. and Desnos, T. (2006) Identification of QTL controlling root growth response to phosphate starvation in Arabidopsis thaliana. Plant, Cell Environ. 29, 115–125. 17086758 10.1111/j.1365-3040.2005.01405.x

[tpj14374-bib-0035] Sawaki, Y. , Iuchi, S. , Kobayashi, Y. ***et al*.** (2009) STOP1 regulates multiple genes that protect arabidopsis from proton and aluminum toxicities. Plant Physiol. 150, 281–294. 19321711 10.1104/pp.108.134700 PMC2675709

[tpj14374-bib-0036] Sawaki, Y. , Kobayashi, Y. , Kihara‐Doi, T. ***et al*.** (2014) Identification of a STOP1‐like protein in Eucalyptus that regulatestranscription of Al tolerance genes. Plant Sci. 223, 8‐15. 24767110 10.1016/j.plantsci.2014.02.011

[tpj14374-bib-0037] Selote, D. , Samira, R. , Matthiadis, A. , Gillikin, J.W. and Long, T.A. (2015) Iron‐binding E3 ligase mediates iron response in plants by targeting basic helix‐loop‐helix transcription factors. Plant Physiol. 167, 273–286. 25452667 10.1104/pp.114.250837 PMC4281009

[tpj14374-bib-0038] Singh, A.P. , Fridman, Y. , Friedlander‐Shani, L. , Tarkowska, D. , Strnad, M. and Savaldi‐Goldstein, S. (2014) Activity of the brassinosteroid transcription factors BRASSINAZOLE RESISTANT1 and BRASSINOSTEROID INSENSITIVE1‐ETHYL METHANESULFONATE‐SUPPRESSOR1/BRASSINAZOLE RESISTANT2 blocks developmental reprogramming in response to low phosphate availability. Plant Physiol. 166, 678–688. 25136063 10.1104/pp.114.245019 PMC4213097

[tpj14374-bib-0039] Singh, A.P. , Fridman, Y. , Holland, N. ***et al*.** (2018) Interdependent nutrient availability and steroid hormone signals facilitate root growth plasticity. Dev. Cell, 46, 59–72. e4. 29974864 10.1016/j.devcel.2018.06.002

[tpj14374-bib-0040] Svistoonoff, S. , Creff, A. , Reymond, M. ***et al*.** (2007) Root tip contact with low‐phosphate media reprograms plant root architecture. Nat. Genet. 39, 792–796. 17496893 10.1038/ng2041

[tpj14374-bib-0041] Thibaud, M.C. , Arrighi, J.F. , Bayle, V. ***et al*.** (2010) Dissection of local and systemic transcriptional responses to phosphate starvation in Arabidopsis. Plant J. 64, 775–789. 21105925 10.1111/j.1365-313X.2010.04375.x

[tpj14374-bib-0042] Ticconi, C.A. , Delatorre, C.A. , Lahner, B. , Salt, D.E. and Abel, S. (2004) Arabidopsis pdr2 reveals a phosphate‐sensitive checkpoint in root development. Plant J. 37, 801–814. 14996215 10.1111/j.1365-313x.2004.02005.x

[tpj14374-bib-0043] Ticconi, C.A. , Lucero, R.D. , Sakhonwasee, S. ***et al*.** (2009) ER‐resident proteins PDR2 and LPR1 mediate the developmental response of root meristems to phosphate availability. Proc. Natl Acad. Sci. USA, 106, 14174–14179. 19666499 10.1073/pnas.0901778106 PMC2723163

[tpj14374-bib-0044] Vert, G. , Grotz, N. , Dedaldechamp, F. ***et al*.** (2002) IRT1, an Arabidopsis transporter essential for iron uptake from the soil and for plant growth. Plant Cell, 14, 1223–1233. 12084823 10.1105/tpc.001388 PMC150776

[tpj14374-bib-0045] Wang, J.J. , Hou, Q.Q. , Li, P.H. ***et al*.** (2017) Diverse functions of multidrug and toxin extrusion (MATE) transporters in citric acid efflux and metal homeostasis in Medicago truncatula. Plant J. 90, 79–95. 28052433 10.1111/tpj.13471

[tpj14374-bib-0046] Wang, X. , Wang, Z. , Zheng, Z. ***et al*.** (2019) Genetic dissection of Fe‐dependent signaling in root developmental responses to phosphate deficiency. Plant Physiol. 179, 300–316. 30420567 10.1104/pp.18.00907 PMC6324241

[tpj14374-bib-0047] Ward, J.T. , Lahner, B. , Yakubova, E. , Salt, D.E. and Raghothama, K.G. (2008) The effect of iron on the primary root elongation of Arabidopsis during phosphate deficiency. Plant Physiol. 147, 1181–1191. 18467463 10.1104/pp.108.118562 PMC2442553

[tpj14374-bib-0048] Wu, W. , Lin, Y. , Chen, Q. ***et al*.** (2018) Functional conservation and divergence of soybean GmSTOP1 members in proton and aluminum tolerance. Front. Plant Sci. 9, 570. 29755502 10.3389/fpls.2018.00570 PMC5932199

[tpj14374-bib-0049] Yamaji, N. , Huang, C.F. , Nagao, S. ***et al*.** (2009) A zinc finger transcription factor ART1 regulates multiple genes implicated in aluminum tolerance in rice. Plant Cell, 21, 3339–3349. 19880795 10.1105/tpc.109.070771 PMC2782276

[tpj14374-bib-0050] Zhang, Y. , Zhang, J. , Guo, J. ***et al*.** (2019) F‐box protein RAE1 regulates the stability of the aluminum‐resistance transcription factor STOP1 in Arabidopsis. Proc. Natl Acad. Sci. USA, 116, 319–327. 30559192 10.1073/pnas.1814426116 PMC6320511

