# Supplementary figures and images for: Under phosphate starvation conditions, Fe and Al trigger accumulation of the transcription factor STOP1 in the nucleus of Arabidopsis root cells
Source: Plant J. 2019 Jun 4;99(5):937–49. doi: 10.1111/tpj.14374 (PMC6852189; doi:10.1111/tpj.14374)

Figure S1

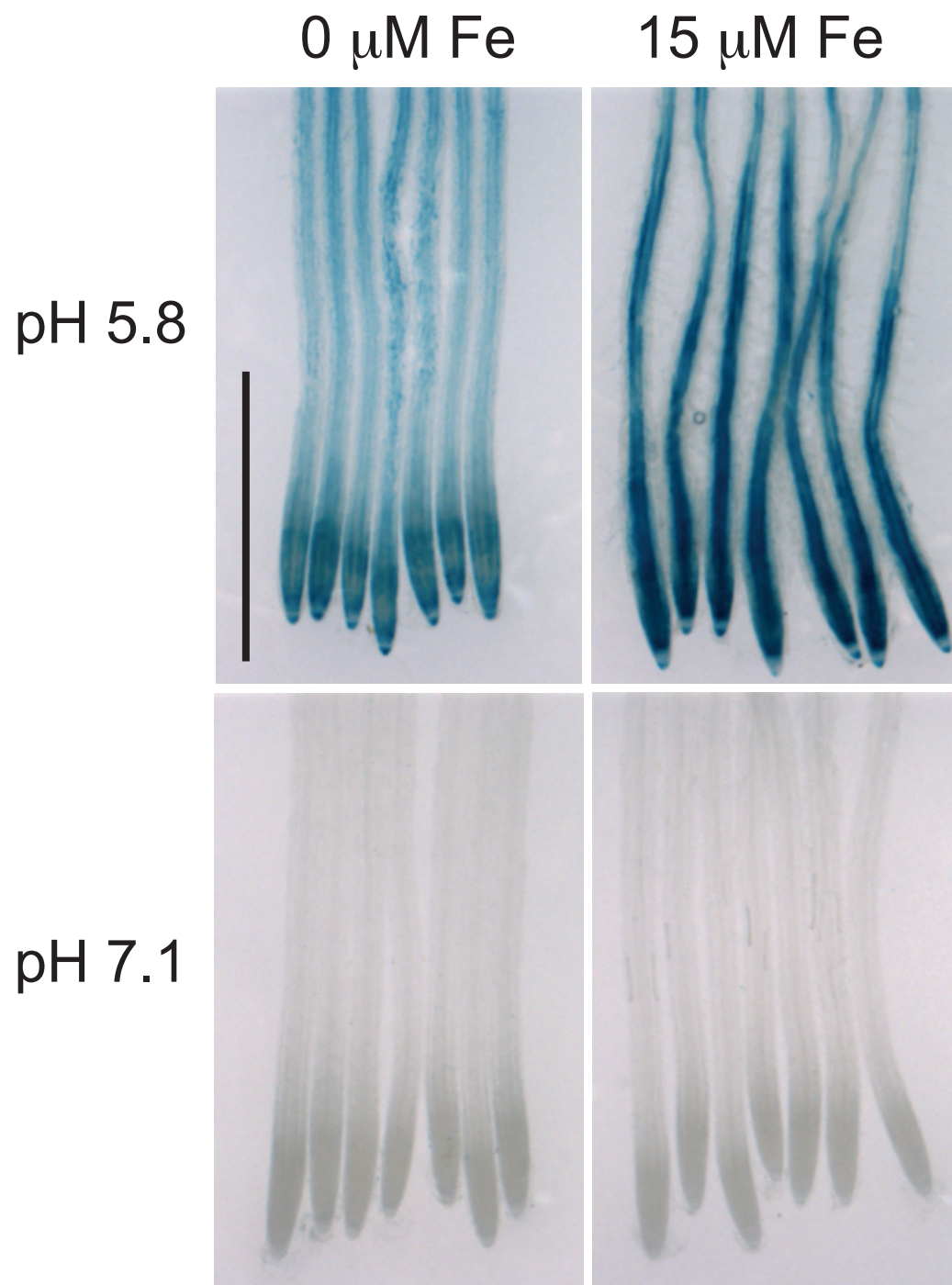

Supplement: Supplementary file 1 — Figure S1. Under neutral conditions, ALMT1 is not expressed. [file TPJ-99-937-s001.pdf]

Figure S2

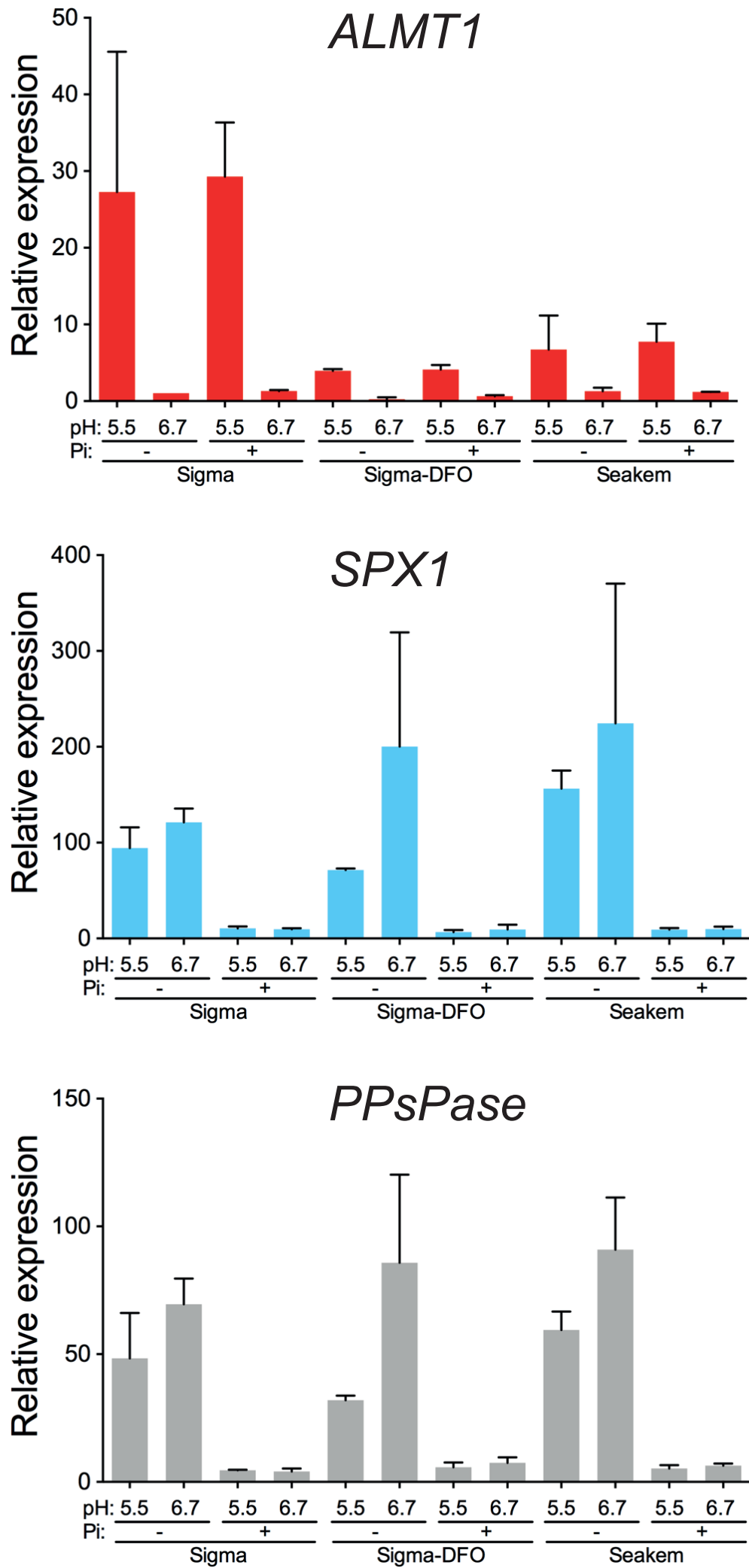

Supplement: Supplementary file 2 — Figure S2. Expression of ALMT1, SPX1 and PPsPase in seedlings roots. [file TPJ-99-937-s002.pdf]

Figure S3

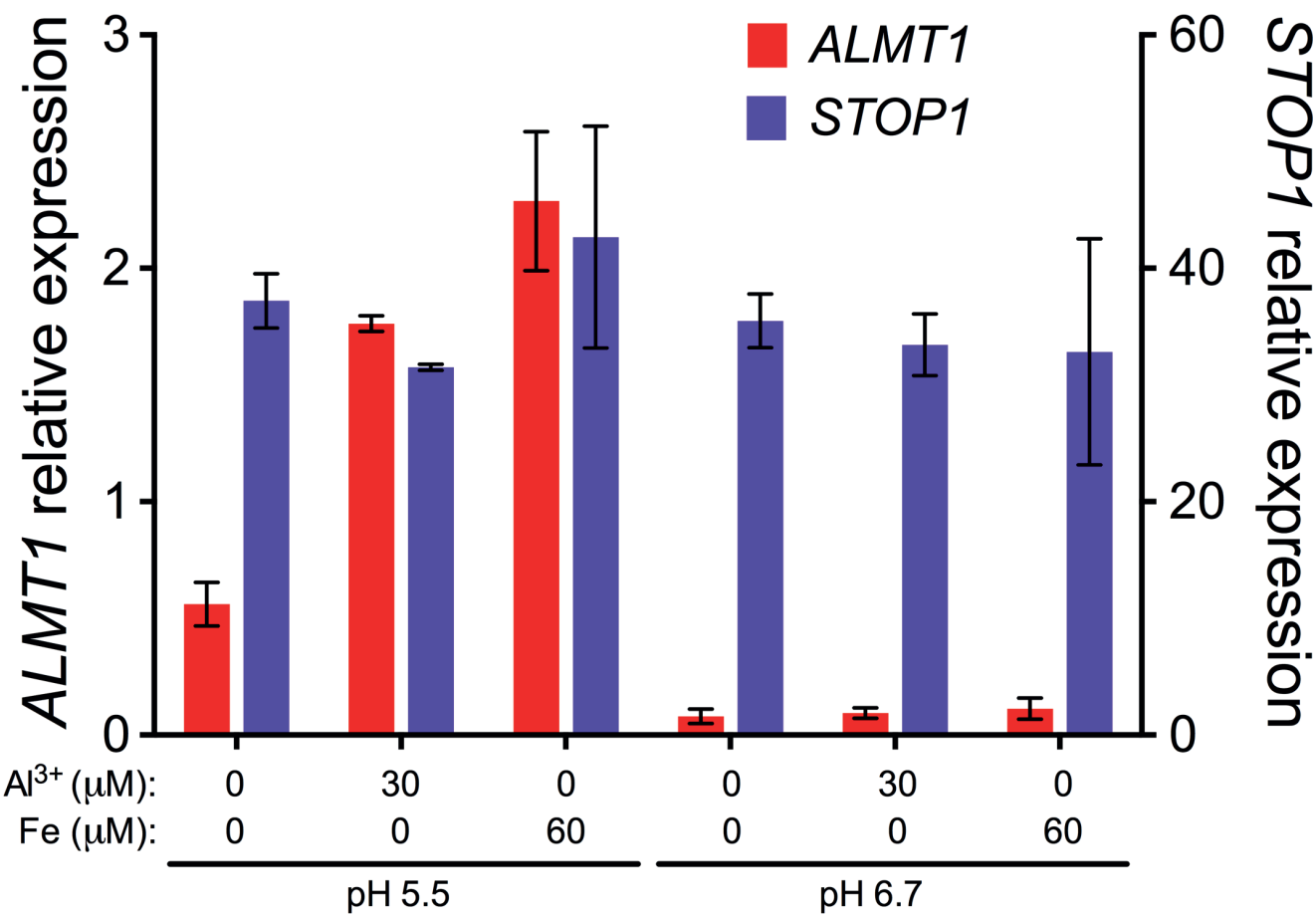

Supplement: Supplementary file 3 — Figure S3. Analysis of STOP1 mRNA expression. [file TPJ-99-937-s003.pdf]

Figure S4

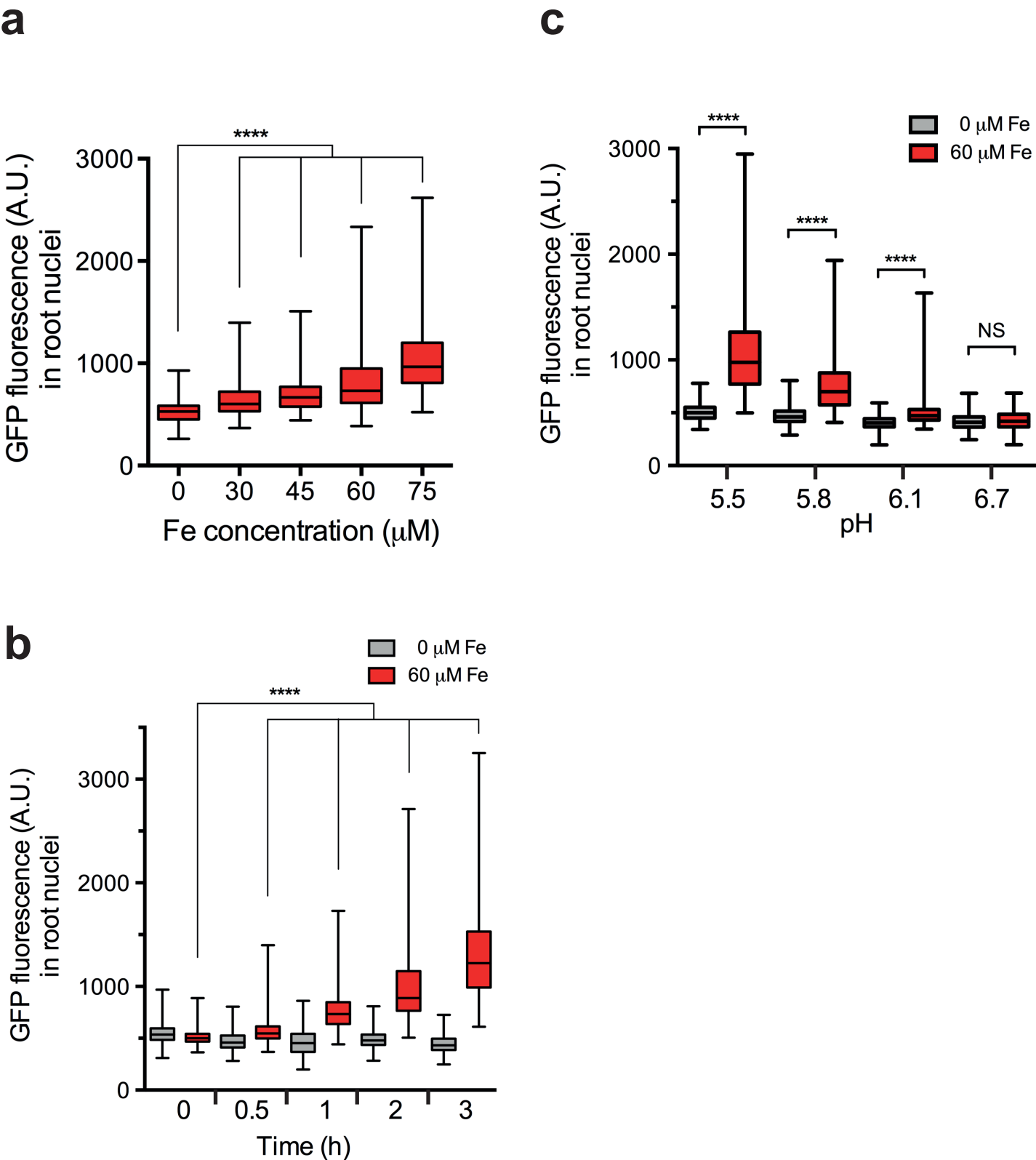

Supplement: Supplementary file 4 — Figure S4. Iron promotes the accumulation of GFP‐STOP1 in root nuclei. [file TPJ-99-937-s004.pdf]

Figure S5

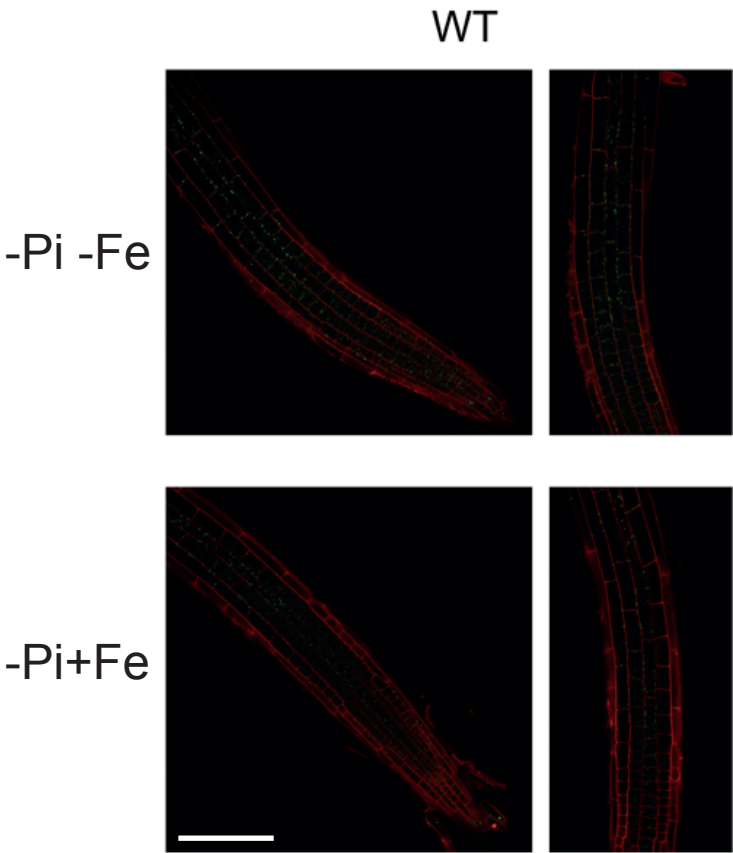

Supplement: Supplementary file 5 — Figure S5. Autofluorescence in the root tip of non‐transgenic wild‐type seedlings. [file TPJ-99-937-s005.pdf]

Figure S6

**a**

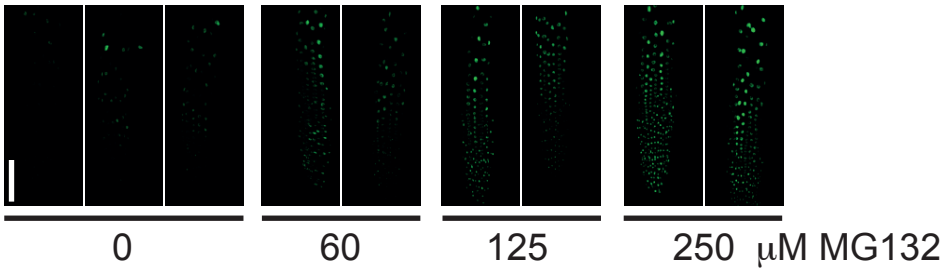

**b**

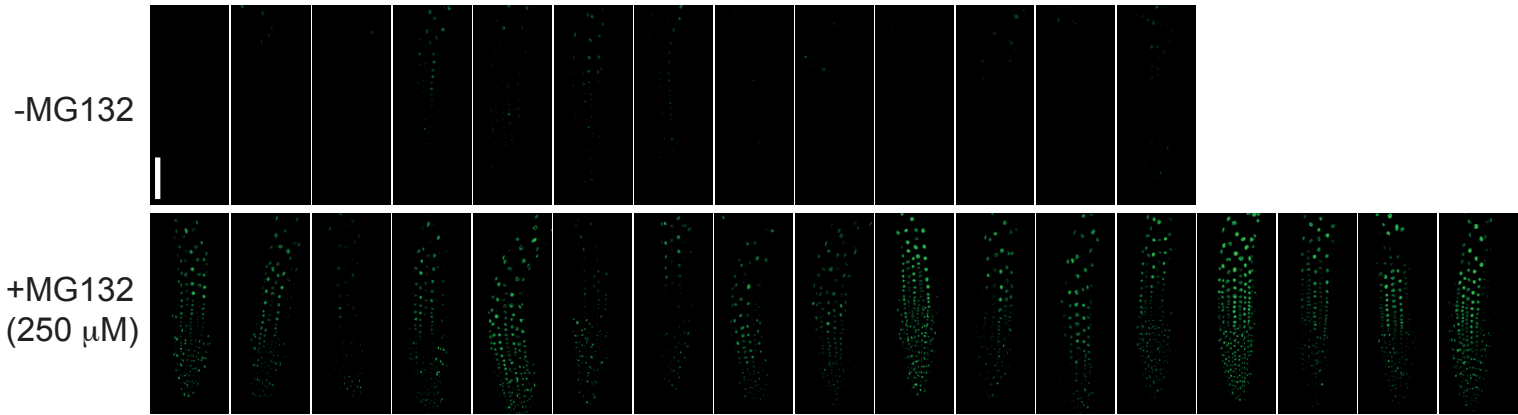

Supplement: Supplementary file 6 — Figure S6. The 26S proteasome inhibitor MG132 promotes accumulation of GFP‐STOP1 in root nuclei. [file TPJ-99-937-s006.pdf]

Figure S7

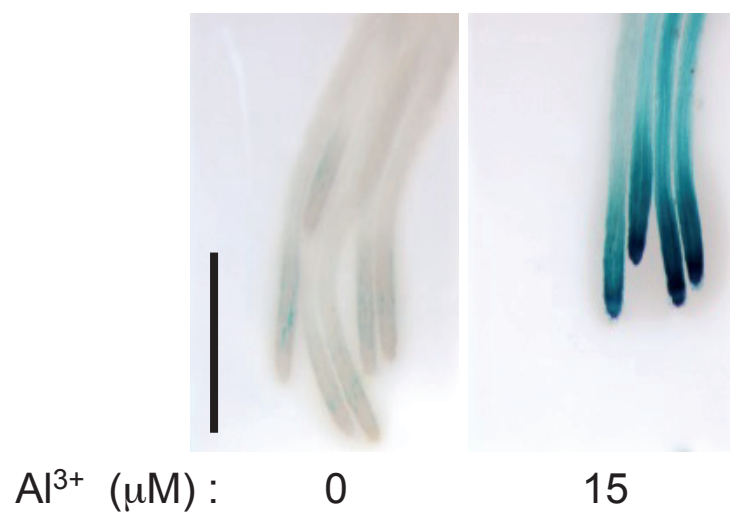

Supplement: Supplementary file 7 — Figure S7. Aluminum stimulates the expression of pALMT1::GUS. [file TPJ-99-937-s007.pdf]

Figure S8

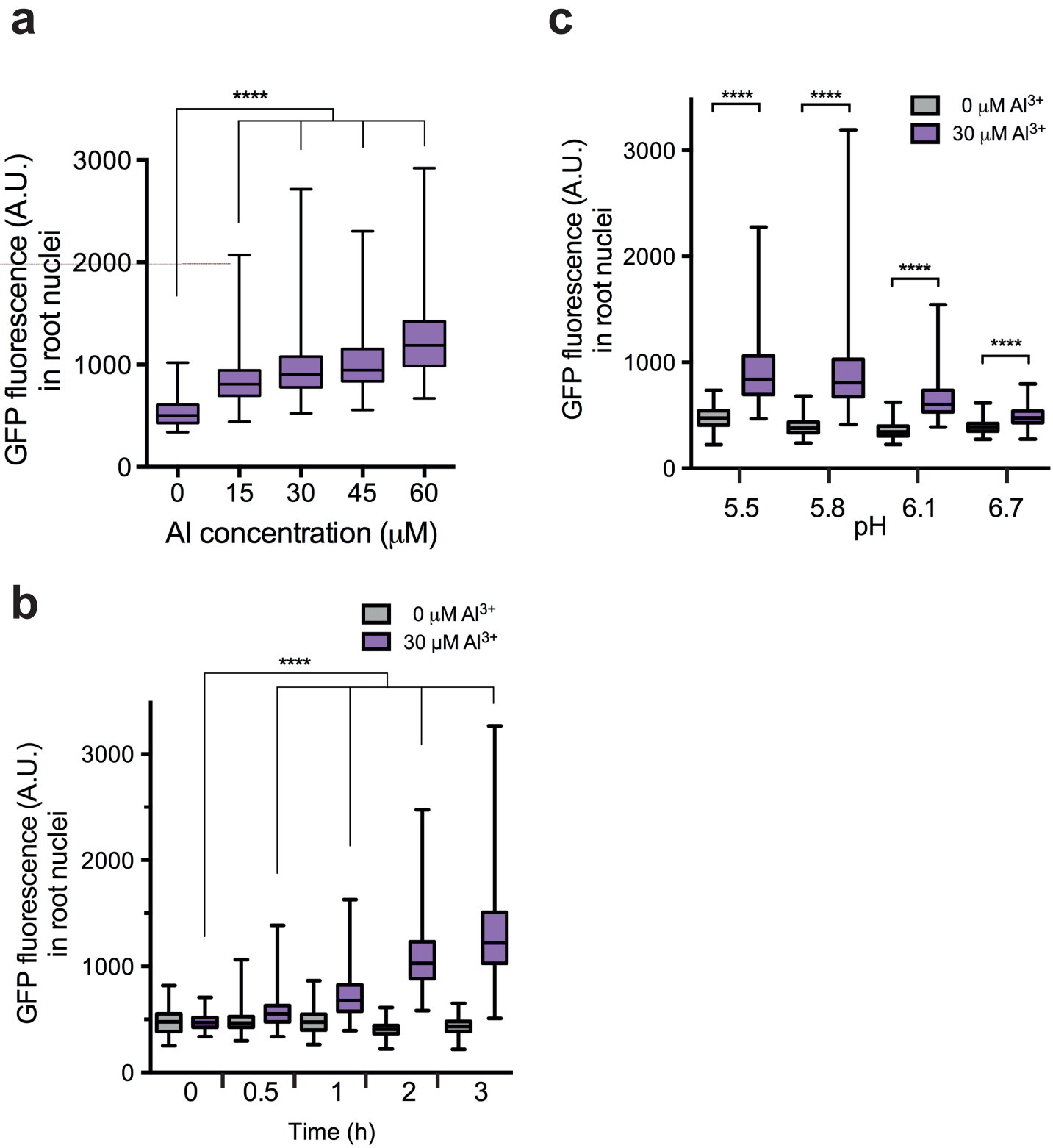

Supplement: Supplementary file 8 — Figure S8. Al3+ promotes the accumulation of GFP‐STOP1 in root nuclei. [file TPJ-99-937-s008.pdf]

Figure S9

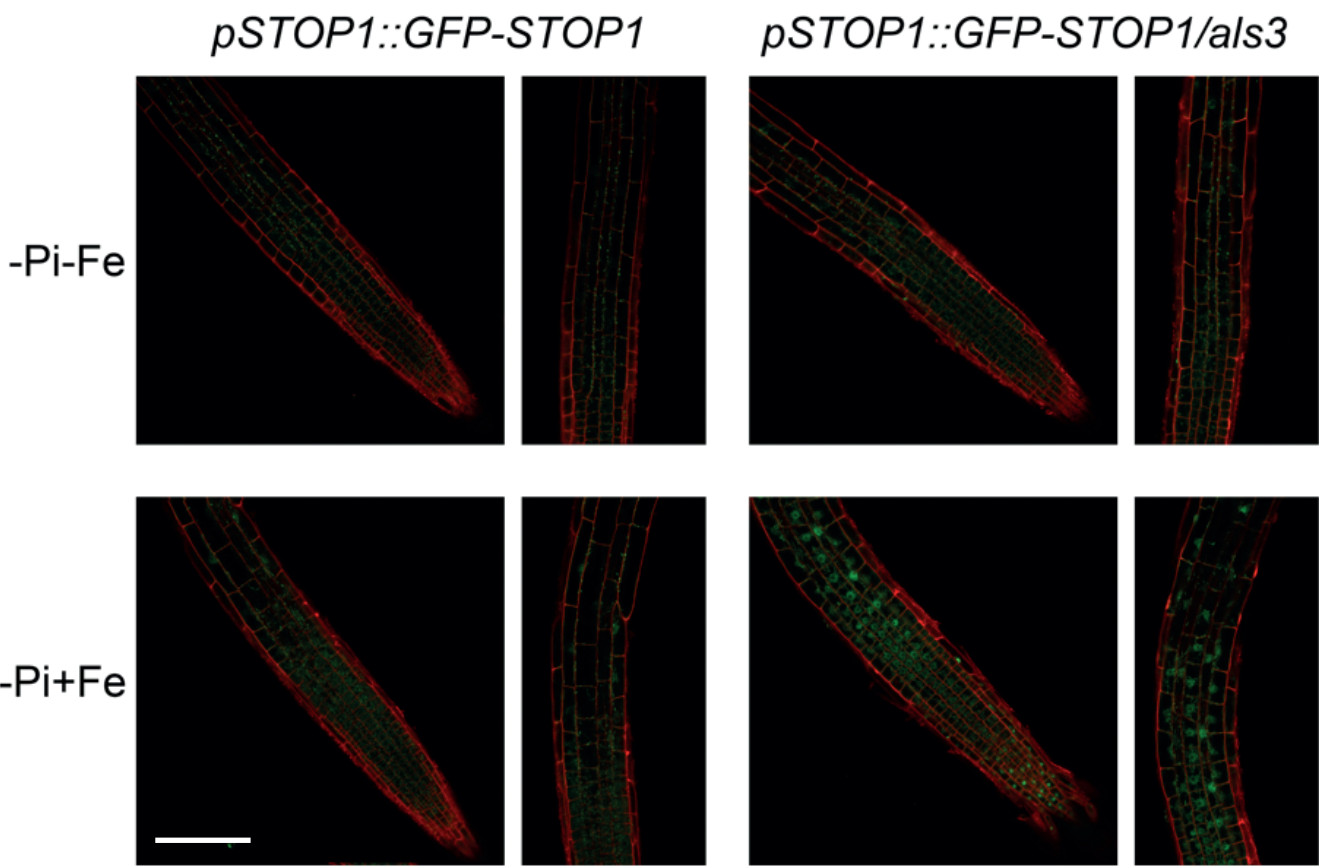

Supplement: Supplementary file 9 — Figure S9. ALS3 represses the accumulation of STOP1 in root nuclei. [file TPJ-99-937-s009.pdf]
