# Supplementary material for: Under phosphate starvation conditions, Fe and Al trigger accumulation of the transcription factor STOP1 in the nucleus of Arabidopsis root cells
Source: Plant J. 2019 Jun 4;99(5):937–49. doi: 10.1111/tpj.14374 (PMC6852189; doi:10.1111/tpj.14374)
Supplement: Supplementary file 10 — Table S1. Aluminum, Fe and P content (μg/100 mg agar or agarose). Table S2. Primer sequences. [file TPJ-99-937-s010.docx]

**Table S1.** Al, Fe and P content (μg/100 mg agar or agarose)

|  | Al | Fe | P |
| --- | --- | --- | --- |
| Agar Sigma A1296 | 5,75 | 3,82 | 5,86 |
| Agar Sigma A1296 treated with DFO and washed | 5,87 | 3,50 | 0,63 |
| Agarose lonza Seakem | 0,42 | 0,24 | 0,81 |

**Table S2.** Primers sequence

| gene | primer | sequence (5’ to 3’) |
| --- | --- | --- |
| *PPsPase1* | AT1G73010_R | GACGACACGTGGATGAATTG |
|  | AT1G73010_F | TCATGATCAAGGCAAAACCA |
| *SPX1* | AT5G20150_R | GCGGCAATGAAAACACACTA |
|  | AT5G20150_F | CGGGTTTTGAAGGAGATCAG |
| *IRT1* | AT4G19690_R | GACGATAGAACTATACTGCCTTGA |
|  | AT4G19690_F | TGCGGAATTGAAATCATGTG |
| *ALMT1* | AT1G08430_R | CGATTCCGAGCTCATTCTTC |
|  | AT1G08430_F | GGCAGTGTGCCTACAGGATT |
| *STOP1* | AT1G34370_F | AAGTGGCTTTGTTCCTGTGG |
|  | AT1G34370_R | GGCTGTGTGGTTTCTTGGTT |
| *Tubulin* | AT5G62690_R | ACACCAGACATAGTAGCAGAAATCAAG |
|  | AT5G62690_F | GAGCCTTACAACGCTACTCTGTCTGTC |
