# Supplementary material for: Under phosphate starvation conditions, Fe and Al trigger accumulation of the transcription factor STOP1 in the nucleus of Arabidopsis root cells
Source: Plant J. 2019 Jun 4;99(5):937–49. doi: 10.1111/tpj.14374 (PMC6852189; doi:10.1111/tpj.14374)
Supplement: Supplementary file 11 [file TPJ-99-937-s011.docx]

**Supporting figure legends**

**Figure S1.**

Picture of the root tip after the GUS staining, of WT seedlings carrying the *pALMT1::GUS* marker. Seedlings were grown in low-Pi at pH 5.8 or 7.1, with or without 15 μM Fe. Note that at pH 7.1 no GUS staining is detected. Bar, 1 mm.

**Figure S2.**

Seedlings were grown 3 days on the indicated media before RNA extraction and qRT-PCR analysis. Mean +/-SD (*n* = two independent experiments).

**Figure S3.**

WT seedlings were pre-grown 3 days under low-Pi condition at pH 5.8 or 7 without Fe or Al added, transferred for 3 hours in the same original pH condition with or without Al^3+^ or Fe^2+^ before extraction of root RNAs and qRT-PCR reactions. The conditions for qRT-PCR reactions and the calculations of relative expression were as in Figure 3 and Figure S2. Mean +/-SD (*n* = two independent experiments). *ALMT1* was used as a control.

**Figure S4.**

The GFP fluorescence was measured (a.u.) in nuclei at the root tip of *pSTOP1::GFP-STOP1* seedlings.

a) Three-day-old seedlings were transferred 2 h in -Pi plates with the indicated concentration of Fe.

b) Three-day-old seedlings were transferred for the indicated time in -Pi plates containing 0 or 60 μM Fe.

c) Three-day-old seedlings were transferred 2h in -Pi plates buffered at the indicated pH, containing 0 or 60 μM Fe.

d) Picture of GFP fluorescence at the root tip, in seedlings transferred 2h in -Pi plates without (left) or with (right) 60 μM Fe.

Box plots indicate the median, the 25^th^ to 75^th^ percentiles (box edges) and the min to max range (whiskers); Mann-Whitney test; ****P<0.0001; NS, not significant (P>0.05); number of nuclei per condition: a) 329-346; b) 291-343; c) 322-392).

**Figure S5.**

Non-transgenic WT seedlings were grown on a -Pi-Fe plate made with the DFO-agar, transferred to a –Pi plate containing 0 or 60 μM Fe for 2 h, and the fluorescence pictured by confocal microscopy (same setting as in Figure S9). Note the small autofluorescent dots. Bar, 100 μm.

**Figure S6.**

Three days old seedlings were treated 6 h (a) or 4 h (b), without or with 60, 125 or 250 μM (a) or 250 μM (b) MG132, and photographed as in Figure 4b. **a** and **b** represent two independent experiments. Bars, 100 μm.

**Figure S7.**

Seedlings were grown four days on a -Pi medium pH 5.5, made with a washed DFO-treated agar, supplemented or not with 15 μM Al^3+^, before GUS staining. Note that the 0 Al control is the same as in Figure 2b, since it is part of the same experiment. Bar, 1 mm.

**Figure S8.**

The GFP fluorescence was measured (a.u.) in nuclei at the root tip of pSTOP1::GFP-STOP1 seedlings.

a) Three-day-old seedlings were transferred 2 h in -Pi plates with the indicated concentration of Al^3+^.

b) Three-day-old seedlings were transferred for the indicated time in -Pi plates containing 0 or 30 μM Al^3+^.

c) Three-day-old seedlings were transferred 2h in -Pi plates buffered at the indicated pH, containing 0 or 30 μM Al^3+^.

Box plots indicate the median, the 25^th^ to 75^th^ percentiles (box edges) and the min to max range (whiskers); Mann-Whitney test; ****P<0.0001; number of nuclei per condition: a) 315-348; b) 330-399; c) 315-448).

**Figure S9.**

a) WT and the *als3* mutant seedlings carrying the *pSTOP1::GFP-STOP1* construct were grown 3 days on a -Pi-Fe plate, transferred to -Pi or -Pi-Fe plates for 2 h, and GFP-fluorescence was pictured by confocal microscopy. Bars, 100 μm.

**Table S1.** Al, Fe and P content (μg/100 mg agar or agarose)

**Table S2.** Primers sequence
